# Preparation and optimization of poly (lactic-co-glycolic acid) rod-shaped particles in nano size range for paclitaxel delivery

**DOI:** 10.3389/fbioe.2022.1103990

**Published:** 2022-12-16

**Authors:** Mengyao Xu, Zuyue Liao, Yang Liu, Shiwei Guo, Haiyang Hu, Tao Chen, Yuesong Wu, Shengli Wan, Meiling Zhou, Muhe Lu, Shiluo Jiluo, Lan Yao, Xiaofeng Pu, Shurong Wang, Qingze Fan

**Affiliations:** ^1^ Department of Pharmacy, The Affiliated Hospital of Southwest Medical University, Luzhou, Sichuan, China; ^2^ Department of Clinical Pharmacy, School of Pharmacy, Southwest Medical University, Luzhou, Sichuan, China

**Keywords:** non-spherical polymeric nanoparticle, rod shape, poly (lactic-co-glycolic) acid, emulsion solvent evaporation method, orthogonal experiment

## Abstract

Nanoparticle shape has been acknowledged as an important design parameter due to its influence on nanoparticle interaction with biological systems. However, there is lacking of simple and scalable preparation technique for drug loaded non-spherical polymeric nanoparticles for a long time, thus hindering the potential applications. Although our previous research has modified the traditional emulsion solvent evaporation technique by adding guest molecules to prepare non-spherical poly (lactic-co-glycolic acid) (PLGA) particles, it is difficult to obtain nano-sized rods with minor axis less than 200 nm, which may have great potential in cancer therapy. Herein, in present research, the two-step ESE method was used and optimized to prepare poly (lactic-co-glycolic acid) nanorods for paclitaxel delivery. Firstly, the single-factor experiment was used to screen the influence of multi-factors including type of guest molecules, concentration of guest molecules, emulsification method, surfactant concentration, oil volume, poly (lactic-co-glycolic acid) concentration on the size and shape to determine the range of variables; based on the above range, a multi-factor and multi-level orthogonal experiment was designed. The formula is evaluated by the rod fabrication yield and the aspect ratio of major axis to minor axis. The results showed that the yield of nanorods in the optimal formula was 99% and the aspect ratio was 5.35 ± 2.05 with the minor axis of 135.49 ± 72.66 nm, and major axis of 657.77 ± 307.63 nm. In addition, the anti-cancer drug paclitaxel was successfully encapsulated in PLGA nanorods by the same technique. Our results not only enrich the ESE technique for preparing small sized poly (lactic-co-glycolic acid) nanorods, but also envision the potential application of nanorods for targeted cancer therapy with the delivery of paclitaxel.

## 1 Introduction

Over the past few decades, biodegradable polymeric particulate systems such as nano/micro spheres have been widely recommended for various drug delivery due to ease of fabrication, stability during storage and delivery, controlled drug release, improved therapeutic effects and fewer side effects (Birk et al., 2021; Jana et al., 2021). It is widely acknowledged that the physiochemical properties such as size, surface chemistry and material composition have important influences on their interaction with biological systems, eventually affecting their therapeutic effects (Yoo et al., 2011; Albanese et al., 2012) However, the effect of shape is usually ignored. Recently, researchers have found that the use of non-spherical drug delivery carriers can be an effective way to overcome certain limitations associated with spherical systems (Lomelí-Rosales et al., 2019). Drug carriers with different shapes can offer different properties in cellular interactions, vascular transport, biodistribution, and immune response (Champion et al., 2007b; Gratton et al., 2008; Barua et al., 2013; Thompson et al., 2013; Gong et al., 2014; Namdee et al., 2014; Safari et al., 2020; Kapate et al., 2021). Champion and Mitragotri (2006) have found that cells can determine whether or not phagocytose a nanoparticle through recognizing the surface shape of the nanoparticle at the initial contact. Meanwhile, non-spherical particles have increased margination (localization and adhesion) to the blood vessel wall in flow (Thompson et al., 2013; Lomelí-Rosales et al., 2019). Similarly, studies suggest that non-spherical particles such as nanorods (Zhou et al., 2013), nanoworms (Wang G. et al., 2014), and nanodisks (Muro et al., 2008) also exhibit longer blood-circulation time and improved tumor accumulation in comparison with their spherical counterpart. Collectively, shape of nanoparticles is increasingly considered as an important parameter in the design of drug delivery carriers due to its significant influence on biological process.

Seeing the aforementioned unique properties of non-spherical shapes, several different shape-engineering techniques have been developed for polymeric nanoparticles which include non-wetting templates, photolithography, microfluidic systems, template assemblies, and film stretching (Gratton et al., 2008; Wang W. et al., 2014; Mathaes et al., 2015; Kapate et al., 2021). Collectively, these methods have produced particles of several distinct shapes. Some of these methods provide advantages such as precise control over particle shape and complex shape preparation. However, they also suffer from drawbacks including complex process of preparation, requirement of special equipment, high cost, and difficult to scale up (Xu et al., 2005; Perry et al., 2011; Wang W. et al., 2014; Cao et al., 2019). Accordingly, lack of simple, versatile, inexpensive, and high-throughput methods for fabricating non-spherical polymeric particles remains a bottleneck in the fields of drug delivery system (Champion et al., 2007a). On the other hand, emulsion solvent evaporation (ESE) method has been considered as a simple, versatile, inexpensive and easy to scale up method to prepare polymeric particles (Heslinga et al., 2009; Rahman et al., 2010). But nanoparticles prepared by the emulsion solvent evaporation method are usually spherical shape due to the spontaneous minimization of interfacial energy for emulsion droplets. Fabrication of non-spherical polymeric nanoparticles by the ESE method remains to be a great challenge. Recently, some researchers have found that, by adding some small guest molecules such as sodium tripolyphosphate (STP), tris base or glycerol, the oil droplets of polymer can be deformed to non-spherical shape in ESE (Heslinga et al., 2009; Li et al., 2010). For example, Omolola Eniola-Adefeso’s group has fabricated rod shaped poly (lactic-co-glycolic acid) (PLGA) microparticles by ESE method with the addition of guest molecule of tris base (Heslinga et al., 2009). Through modifying the fabrication method with two-step ESE method, rod-shaped PLGA submicron particles with minor axis down to 700 nm are successfully obtained (Safari et al., 2018). However, this size of nanoparticle is still too large when applied in tumor targeted therapy.

Previously, we also fabricated the non-spherical PLGA particles by the ESE method (Fan et al., 2016). A more commonly used inorganic salt, phosphate buffer saline (PBS) was adopted by us as the guest molecules. However, the rod-shaped particles were still in micro size range and the guest molecule PBS was mixed compounds, which made our system more complicated to optimize and hard to elucidate the underlying mechanism. Moreover, several formulation and process variables have great effects on the shape and size of the prepared particles. Researchers can identify the influence of parameters on particle deformation by changing one variable at a time, also defined as a single parameter optimizing method to obtain the optimal formula for rod-shaped nanoparticles. However, when associated with multiple variables, this single parameter method usually consumes a lot of time, money and effort and even fails. Meanwhile, the multi-factor and multi-level orthogonal experiment design is widely used to analyze the influence of multiple variables at the same time with decreased number of experiments (Mensah et al., 2019; Rafiei and Haddadi, 2019). Each variable can be assessed independently of all other variables so that it is more effective and economic than conventional experimental methods (Du et al., 2020).

Herein, we show that with the addition of the single guest molecule disodium hydrogen phosphate (Na_2_HPO_4_), the deformed PLGA particles can be obtained by ESE technique. By combining the ultrasonication method with two-step ESE technique, smaller PLGA nanorods with the minor axis down to 200 nm can be successfully prepared. Moreover, the influence of process parameters including ultrasonic power, Na_2_HPO_4_ concentration, surfactant concentrations, oil volume and PLGA concentration on the fabrication yield and aspect ratio of nanoparticles was comprehensively investigated by the orthogonal experiment method and the optimal combination of factor levels was achieved (Mensah et al., 2019; Rafiei and Haddadi, 2019; Du et al., 2020). Finally, the anti-cancer drug paclitaxel was successfully encapsulated in PLGA nanorods by the same technique.

## 2 Materials and methods

### 2.1 Materials

PLGA copolymers with lactic: glycolic acid ratios of 75:25 (Molecular weight (MW): 17–20 kDa) were purchased Daigang Biological Engineering Co., Ltd., (Shandong, China). Paclitaxel (PTX, degree of purity ≥99%, BR) was procured from Dalian Meilun Biotechnology Co., Ltd., China. Dichloromethane was procured from Sichuan Fairbest Technology Co., Ltd., China. PBS was purchased from Solarbio Biological Technology Co., Ltd., China. Sodium Chloride (NaCl), Sodium Dihydrogen Phosphate Anhydrous (NaH_2_PO_4_) and Acetone were analytical reagent (AR) and purchased from Chengdu Kelong Chemical Co., Ltd., China. Acetonitrile (chromatographic grade) was purchased from Chengdu Kelong Chemical Co., Ltd., China. Disodium hydrogen phosphate (Na_2_HPO_4_) (AR) was purchased from Tianjin Zhiyuan Chemical Reagent Co., Ltd., China. Polyvinyl alcohol (PVA, PVA-217, degree of polymerization 1700) was purchased from Shanghai Yingjia Industrial Development Co., Ltd., China.

### 2.2 Methods

#### 2.2.1 One-step preparation of PLGA micro-rods

Firstly, the conventional one-step emulsion solvent evaporation technique was used to develop PLGA non-spherical nanoparticles and the flow chart of one-step method is shown in Supplementary Figure S1A. Briefly, PLGA was dissolved into dichloromethane as the oil phase. Subsequently, the oil phase was added to the water phase which was consisted of a certain amount of PVA and guest molecules (PBS and other inorganic salts) under stirring. And the formed oil-in-water emulsion was stirred at 500 rpm for at least 4 h on a magnetic stirrer (RT 10, IKA, Germany) for particle solidification. Finally, the formed nanoparticles were observed under an inverted light microscope.

#### 2.2.2 Two-step preparation of PLGA nanorods

In this experiment, the fabrication of biodegradable PLGA non-spherical nanoparticles was completed by a two-step method The flow chart of two-step method is shown in Supplementary Figure S1B. First of all, the PLGA oil phase was poured into PVA 1. Then, an O/W emulsion was made on an ultrasonic homogenizer (SONOPULS HD4100, BANDELIN, Germany) in an ice bath for 2 min (the first step for emulsion formation). Subsequently, the O/W emulsion was poured into PVA 2 which contained the guest molecules of Na_2_HPO_4_ for droplets stretching and deforming (the second step for emulsion deformation). The concentration of PVA 1 was lower than the concentration of PVA 2. And the emulsion was solidified with magnetic stirring at 500 rpm for at least 4 h at room temperature to allow for sufficient evaporation of the organic solvent. The resultant nanoparticles were collected and washed with distilled water for six times in a high-speed centrifuge (40,000 g, 10 min) (LYNX6000, Thermo Scientific, America) and subsequently stored at −20°C until further use. Scanning electron microscopy (SEM) (JEM -6700F, JEOL, Japan) was used to observe the size and morphology of PLGA nanorods. Specifically, fabricated nanoparticles were suspended in deionized water and naturally dried on a clean silver paper. Then, dried particles were sprayed with gold before SEM imaging.

### 2.3 Experimental design

#### 2.3.1 The single-factor experiment

At the beginning of optimizing the two-step ESE process for nanorods, the single-factor experiment design was used to explore the influence of multi-factors including emulsification method, ultrasonic power, guest molecule concentration of causing particles deformation, PVA concentrations oil volume and PLGA concentration on the size and shape to determine the range of variables. The factors were selected based on the previous researches (Fan et al., 2016; Safari et al., 2018). Supplementary Table S1 lists the experimental scheme. The deformation of PLGA nanoparticles was observed by SEM.

#### 2.3.2 The multi-factors and multi-levels orthogonal experiment

Based on the range of the single-factor experiment, a multi-factor multi-level orthogonal experiment was designed (Table 1). Six factors including ultrasonic power, Na_2_HPO_4_ concentration, PVA 1 concentration in the first aqueous phase, PVA 2 concentration in the second aqueous phase, oil volume and PLGA concentration were evaluated at three levels. When each of the six parameters was studied at three levels, the number of experiments required for full factorial design could reach 729 (=3^6^), which was too large to be fulfilled in a limited time and at acceptable test expenses. In the multi-factor multi-level orthogonal experiment, however, the number for the six-factor three-level case could be significantly reduced to 18 according to an L_18_ (3^6^) orthogonal table, which obviously alleviates the workload compared to 729 in the full factorial design schemes. Supplementary Table S2 lists 18 different combinations of factor levels based on the orthogonal arrangement principle with the help of the software “IBM SPSS Statistics.” The formula was evaluated by the rod fabrication yield and the aspect ratio of major axis to minor axis. Major axis and minor axis of PLGA nanorods were measured by the software ImageJ (ImageJ, National Institutes of Health) under the images of SEM, and the number of nanorods measured was more than 50. Nanorods were defined as nanoparticles that had the aspect ratio greater than 2. So, the rod fabrication yield was defined as the percentage of nanoparticles with the aspect ratio greater than 2 in an individual experiment.

**TABLE 1:** Variables (factors) along with their corresponding levels in the orthogonal experiment design for fabrication of PLGA nanorods by two-step ESE method.

Parameters	Factors	Units	Levels		
			Low	Medium	High
A	Na_2_HPO_4_ conc^a^	mM	10	75	150
B	Ultrasonic power	W	40	80	120
C	Oil volume	ml	.5	1.0	1.5
D	PLGA conc^a^	mg/ml	10	20	40
E	PVA 1 conc^a^	wt%	.1	.5	1.0
F	PVA 2 conc^a^	wt%	1.0	2.0	3.0

^a^ Conc, concentration.

The effect of each factor on the aspect ratio and rod fabrication yield PLGA nanorods was evaluated based on K values and range analysis of *R* values, which were the reference standard values and equations for K and R of the orthogonal test were described as follows (Du et al., 2020):

$$K_{i}=S_{i}/S\left(i=1,\;2,\;3\right)$$ (1)

$$R=\mathrm{Max}\left\{K_{1\;},K_{2},K_{3}\right\}-\mathrm{Min}\;\left\{K_{1\;},K_{2},K_{3}\right\}$$ (2)

where S_i_ indicated the sum of the test results corresponding to the *i*th level number (i = 1, 2, 3 in the six-factor three-level case) and S was the number of occurrences of each level in the orthogonal test (S = 6 according to Supplementary Table S2); The R value represented the difference between the maximum and minimum K value of the specific factor, reflecting the significance of the studied factor on the fabricated PLGA nanoparticles. Based on the K and *R* values, the effect and significance of each influential parameter on the aspect ratio and rod fabrication yield of PLGA nanoparticles could be obtained, and thereby the reasonable combinations of factor and level could be proposed to achieve the optimal nanorods with large aspect ratio and high yield.

### 2.4 Preparation and characterization of paclitaxel loaded PLGA nanorods

#### 2.4.1 Preparation

Based on the optimized combination, paclitaxel was added into the oil phase to prepare paclitaxel loaded PLGA nanorods. The preparation methods were the same as Section 2.2.2.

#### 2.4.2 Characterization

The size and morphology of the paclitaxel loaded PLGA nanoparticles were detected by SEM and the major axis, minor axis and the aspect ratio were detected and calculated from SEM images with ImageJ software. The drug loading efficiency (LE) and encapsulation efficiency (EE) of the paclitaxel in PLGA nanorods were evaluated through high-performance liquid chromatography (HPLC) detection (Le Broc-Ryckewaert et al., 2013). Briefly, 2 mg of the freeze-dried drug-loaded nanorods were dissolved in 1 ml of acetonitrile. The mixture was vortexed for 1 min, sonicated for 30 min, and filtered by organic filter head. The concentration of the paclitaxel was then quantified HPLC (Agilent, USA) with the following conditions: column: Kromasil 100-5-C18 (5 μm × 4.6 mm × 150 mm) chromatographic column; mobile phase: the ultrapure water and acetonitrile (40:60, V/V); flow rate: 1 ml/min; detection wavelength: 227 nm; column temperature: 30°C ± 1°C and injection volume: 20 μl.

Then, the drug loading efficiency and encapsulation efficiency of the preparation were calculated as follows:

$$LE\;\%=\frac{weight\;of\;PTX\;in\;particles}{weight\;of\;particles}\times 100\%$$ (3)

$$EE\;\%=\frac{weight\;of\;PTX\;in\;particles}{initial\;weight\;of\;PTX\;added}\times 100\%$$ (4)

## 3 Results and discussion

### 3.1 Screening of guest molecules

Our previous research had proved that PBS could be used as the guest molecule (also described as the deformation inducer) to induce the deformation of emulsion droplets and acquire rod-shaped microparticles (Fan et al., 2016). However, PBS was a mixed salt, containing NaH_2_PO_4_, Na_2_HPO_4_ and NaCl and there was no systematic study on the main compound that cause deformation. Therefore, the amounts of Na_2_HPO_4_, NaH_2_PO4 and NaCl contained in 5 mM PBS were added to the aqueous phase respectively to assess their capacity to induce deformation. As shown in Supplementary Figure S2, both PBS and Na_2_HPO_4_ could induce particle deformation, while the other components did not change the spherical morphology of particles. Therefore, it could be concluded that the Na_2_HPO_4_ was the main component in PBS that caused microparticle deformation. Compared with the previously used deformation inducer PBS which was a mixed compound, the inorganic compound Na_2_HPO_4_ was simple and pure and its concentration could be accurately adjusted, thereby benefited for optimizing the effect of its concentration on deformation and clarifying the mechanism. Therefore, in our present system, the formulation of non-spherical PLGA nanoparticles would be carefully and comprehensively optimized with Na_2_HPO_4_ as the guest molecule/deformation inducer.

### 3.2 Preparation and optimization of PLGA nanorods by single parameter method

Although rod-shaped PLGA particles could be prepared by one-step ESE method with the deformation inducer Na_2_HPO_4_, the size of deformed particles prepared was still large in the micron range. Microrods were not conducive to be used as the drug carriers for intravenous administration and might cause serious problems such as vascular embolism. On the other hand, a large number of studies had shown that nanoparticles with a size of <200 nm could be passively targeted to tumor tissue through the enhanced permeation and retention (EPR) effect (Duan and Li, 2013; Hoshyar et al., 2016). Therefore, further optimizing the formulation to obtain nanorods with a short diameter of about 200 nm was the key to solve the problems existing in intravenous injection and tumor targeted therapy.

Previously, the capillary number (Ca) and the viscosity ratio of the dispersed phase to continuous phase (M) were used in our research to explain the possibility and ease of deformation of the emulsion droplets in one-step ESE with the deformation inducer PBS (Fan et al., 2016). Capillary number, also known as interfacial tension number, was a dimensionless concept, which represented the ratio of fluid viscosity force to fluid interfacial tension and could be calculated as follows:

$$Ca={\gamma \eta }_{s}\alpha /\Gamma$$ (5)

$$M=\eta_{d}/\eta_{s}$$ (6)

where *γ* was the shear rate, *α* was the initial droplet size, and *Γ* was the interfacial tension between the dispersed phase and continuous phase, η_d_ and η_s_ were the viscosity of the dispersed phase and continuous phase, respectively. In general, a high Ca value (high viscous forces or low interfacial tension) and/or a low M value result in emulsion conditions that favored droplet deformation. Intermediate values of the viscosity ratio (M ≈ 1) at a fixed capillary number led to severe droplet breakup and high values of M result in no droplet deformation or breakup (Heslinga et al., 2009; Murphy et al., 2020). In order to obtain smaller rods, the size of the initial droplets needed to be very small, which meant a low *α* value. However, decreasing *α* would reduce the Ca value, which then made the deformation process difficult to occur. Therefore, in one-step ESE method, there was contradiction between the formation of smaller droplets and the deformation of droplets. Indeed, the rod-shaped particles fabricated in our previous research by the one-step ESE method were limited to a micron size range (Fan et al., 2016).

Surprising, the research group of Omolola Eniola-Adefeso modified the ESE and found that the contradiction between initial droplet size and droplet tensile deformation could be balanced by changing from one-step method to two-step method (Safari et al., 2018). In other words, by using two different PVA concentrations in the droplet formation step (PVA 1 concentration used) and stretching step (PVA 2 concentration used), a lower *α* and higher Ca/lower M could be simultaneously acquired because the deformation step was temporarily separated from the droplet formation step. After optimization, rod-shaped PLGA particles with the submicron size range were successfully prepared. However, because the emulsion was prepared with the overhead mixer like homogenization, the size of the rods was still large for tumor targeted therapy.

Based on the above analysis, the two-step ESE was adopted with Na_2_HPO_4_ as the deformation inducer to prepare smaller nanorods. A single-factor experimental design was used to investigate the effects of parameters including emulsification method, ultrasonic power, concentration of deformation inducer Na_2_HPO_4_, oil volume, PLGA concentration, and PVA concentration (PVA 1 concentration and PVA 2 concentration) on deformation for further orthogonal experimental optimization.

#### 3.2.1 Effect of emulsification method

**FIGURE 1:**
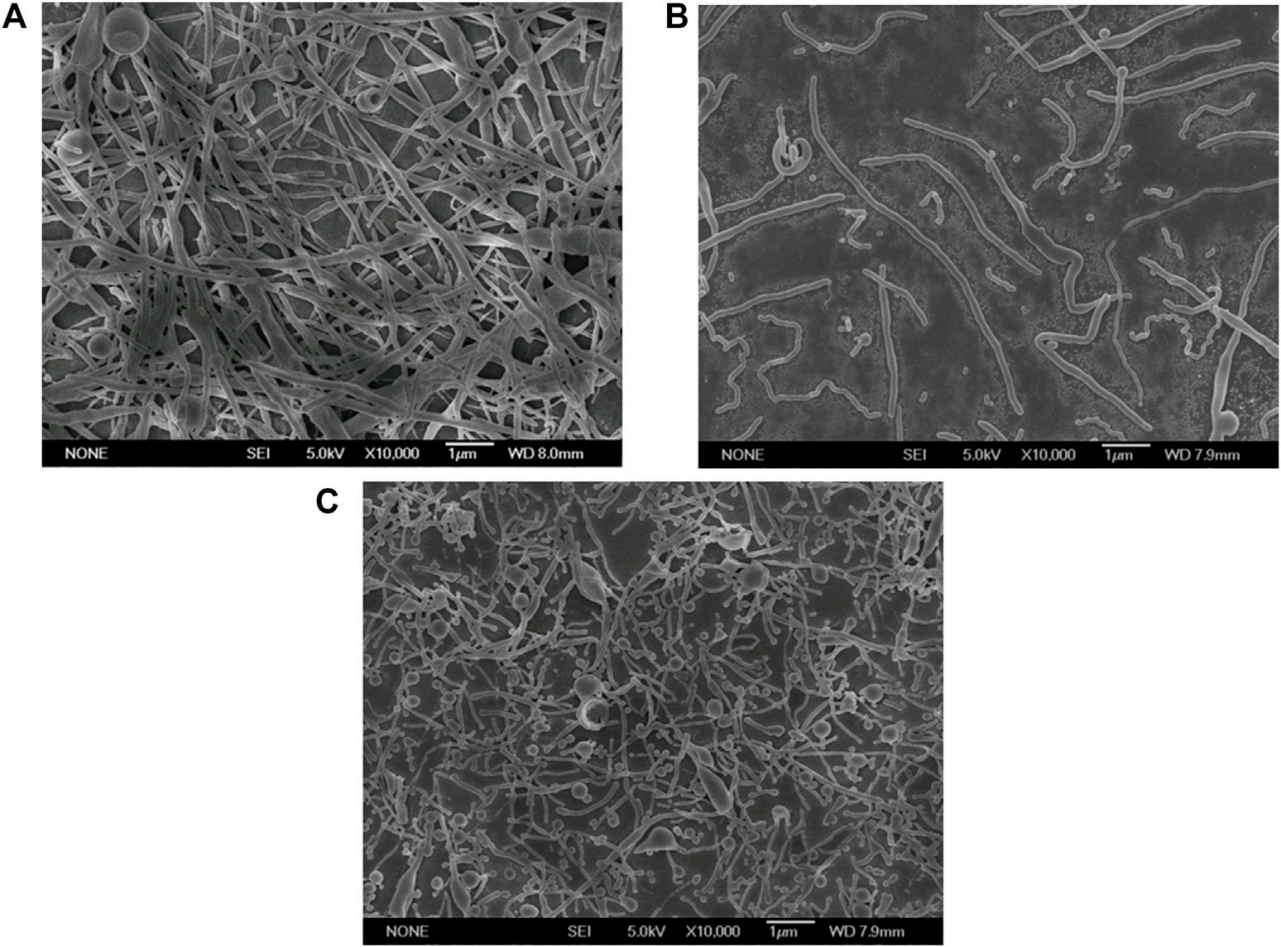
Electron micrographs of PLGA nanoparticles prepared by different emulsification methods (Scale bar: 1 μm). **(A)** Magnetic stirring; **(B)** Homogenization; **(C)** Ultrasonication.

In the ESE, the non-spherical droplets were deformed from the spherical droplets. Therefore, the initial size of the droplet plays a decisive role in the size of the final rods. The smaller the initial particle size, the smaller the rods after stretching theoretically. At the same time, the initial droplet diameter also affected the Ca value. So, it was a key step to explore an appropriate emulsification method so that the droplets were in nano size range and could also effectively deformed. Therefore, we first investigated the influence of three emulsification methods, namely magnetic stirring method, homogenization method and ultrasonic method, on the size and shape of nanoparticles. As shown in Figure 1, the size of the non-spherical particles prepared by magnetic stirring method, homogenization method and ultrasonic method gradually decreased, which could be attributed to the reduced size of the initial emulsion fabricated by ultrasonic method (Supplementary Figure S3).

Therefore, further optimization by ultrasonic technique was expected to obtain ultra-small nanorods.

#### 3.2.2 Effect of ultrasonic power

**FIGURE 2:**
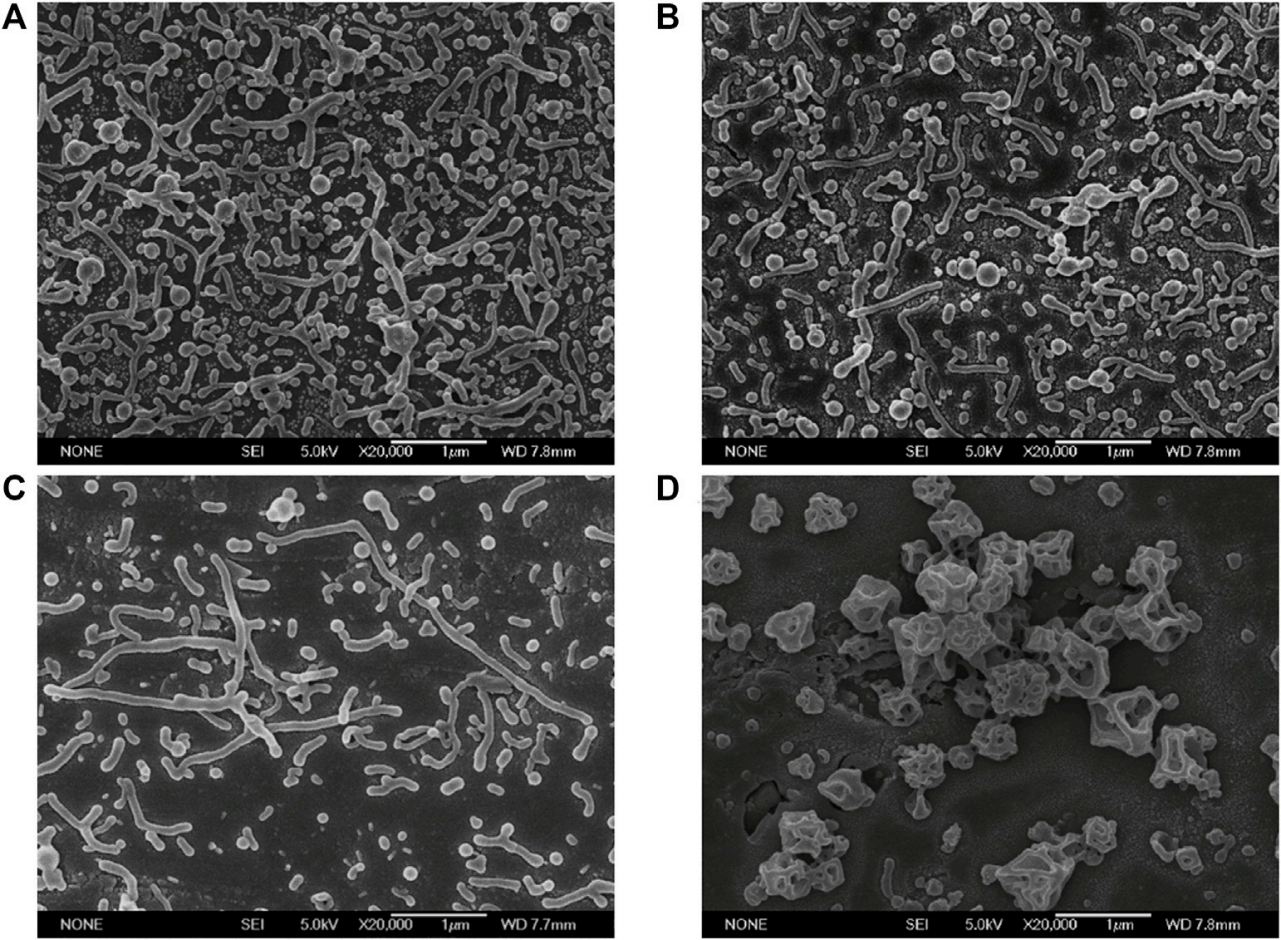
Electron micrographs of PLGA nanoparticles fabricated at different ultrasonic powers (Scale bar: 1 μm). **(A)** 40 W; **(B)** 80 W; **(C)** 120 W; **(D)** 160 W.

In this experiment, four ultrasonic powers were respectively set as 40, 80, 120, and 160 W. As shown in Figure 2, when the ultrasonic power was 40 and 80 W, many of the nanoparticles were deformed to short and small rods; Subsequently, when the ultrasonic power increased to 120 W, the nanoparticles deformed almost completely but were not uniform, with some long rods and short rods. However, when the ultrasonic power was further increased to 160 W, the nanoparticles prepared were irregular bulk particles. Theoretically, the initial droplet size decreased with ultrasound power increasing. Our results showed that, within a certain ultrasonic power range, the deformability and the size of the nanorods had no obvious difference; When the ultrasonic power increased to a certain value (160 W), larger bulk nanoparticles appeared after stretching and solidifying. This phenomenon could be explained by the capillary number. When the ultrasonic power was too large, the initial droplets were too small, so the capillary number decreased and the deformed nanoparticles also decreased. Besides that, nanoparticles prepared by high sonication power were not uniform and the droplets collided with each other during the stretching phase, so the small droplets converged into large droplets to form the irregular bulk particles.

#### 3.2.3 Effect of Na_2_HPO_4_ concentration

**FIGURE 3:**
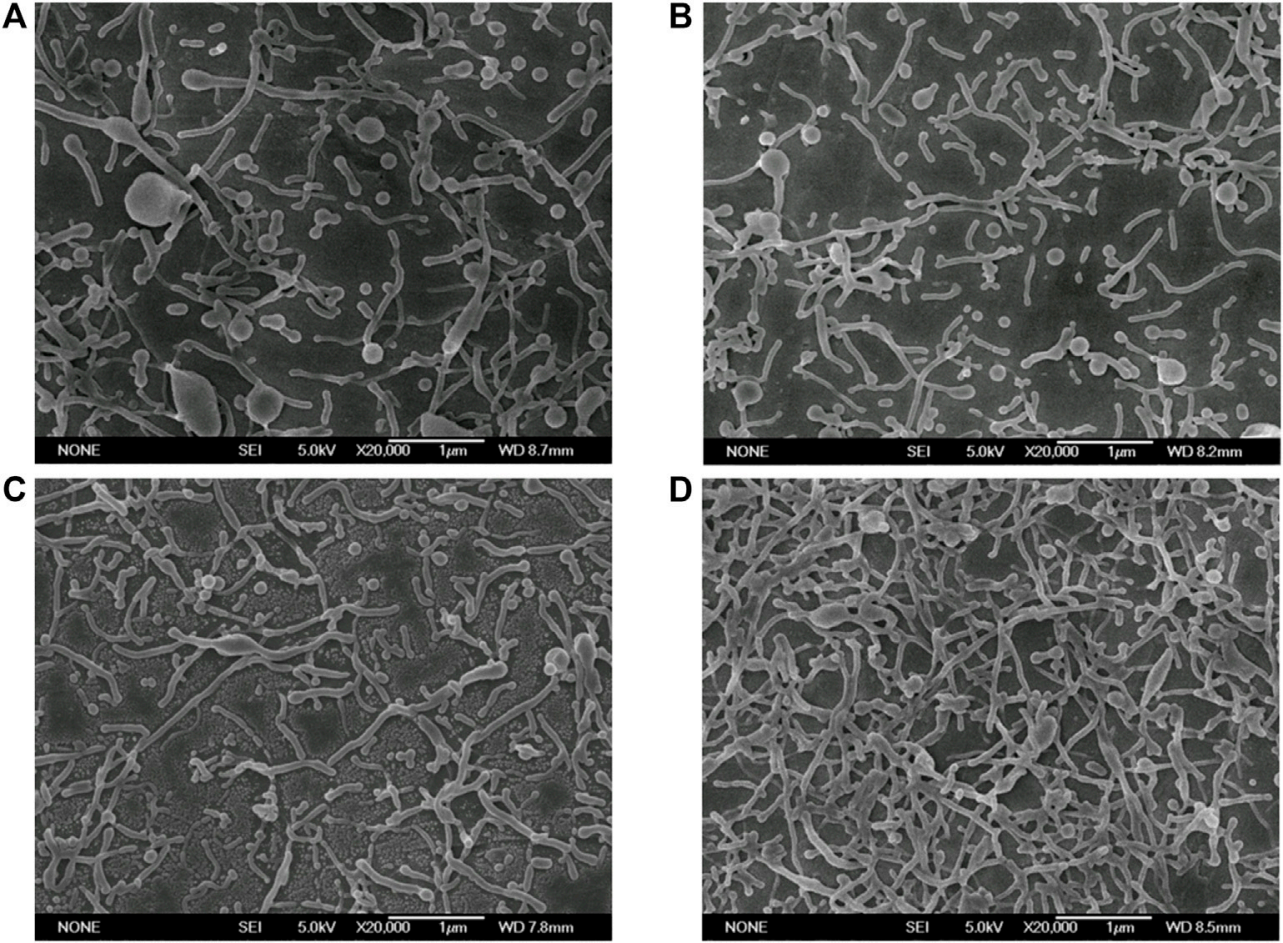
Electron micrographs of PLGA nanoparticles prepared with different concentrations of Na_2_HPO_4_. (Scale bar: 1 μm). **(A)** 50 mM; **(B)** 100 mM; **(C)** 150 mM; **(D)** 200 mM.

As indicated in the one-step ESE results, disodium hydrogen phosphate is the key substance causing the deformation of nanoparticles. As a consequence, four concentrations of Na_2_HPO_4_, 50, 100, 150, and 200 mM, were experimentally examined for their effect on nanoparticle deformation. As shown in Figure 3, different concentrations of disodium hydrogen phosphate could cause different degrees of deformation of nanoparticles. In detail, when Na_2_HPO_4_ was 50 mM, there were many undeformed spherical nanoparticles in the SEM images. When the concentration of Na_2_HPO_4_ increased to 100 mM, most of the nanoparticles were deformed. The deformed nanoparticles were maximum in the concentration of 150 and 200 mM. So, it could be deduced that the deformation degree of the nanoparticles increased as the concentration of deformation inducer increased.

#### 3.2.4 Effect of PLGA concentration

**FIGURE 4:**
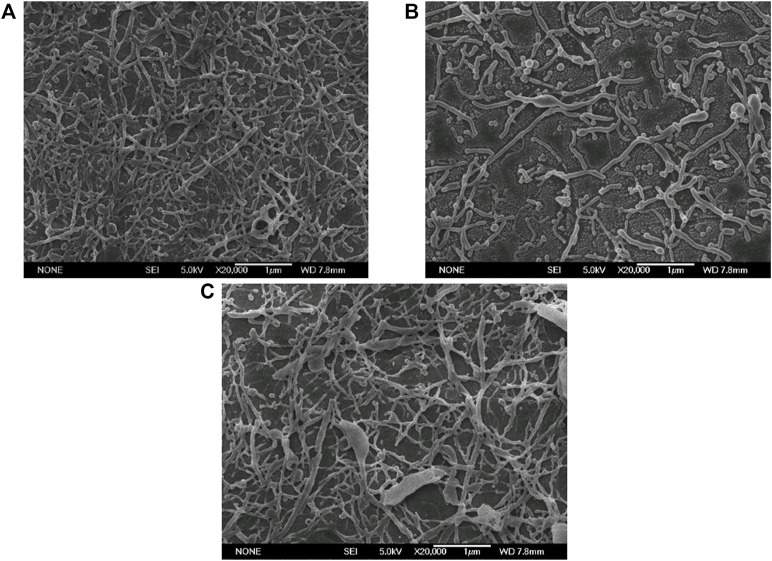
Electron micrographs of PLGA nanoparticles fabricated with different PLGA concentrations (Scale bar: 1 μm). **(A)** 10 mg/ml; **(B)** 20 mg/ml; **(C)** 40 mg/ml.

Three PLGA concentrations, namely 10, 20, and 40 mg/ml, were experimentally set up to explore the effect on PLGA nanoparticles. As shown in Figure 4, when the PLGA concentration was lower than 20 mg/ml, the nanoparticles were uniform with rod shape. When the PLGA concentration increased to 40 mg/ml, many nanoparticles were still in the similar thin and rod shape, but a few large nanorods occured. The PLGA concentration could affect the viscosity of the oil phase. The three concentration investigated were relatively lower than the normal concentration used for preparing nanospheres by ESE (Baghaei et al., 2015; Mensah et al., 2019), so the M value was relatively low and the droplets were all deformed into nanorods. However, under the same sonication power, higher viscosity of the oil phase would be harmful to the uniformity of the droplets, thereby some large nanorods were fabricated with 40 mg/ml. Comprehensively consider the relationship between PLGA concentration, particle size and amount of nanorods, PLGA concentration was chosen as 20 mg/ml for the subsequent investigation.

#### 3.2.5 Effect of oil volume

**FIGURE 5:**
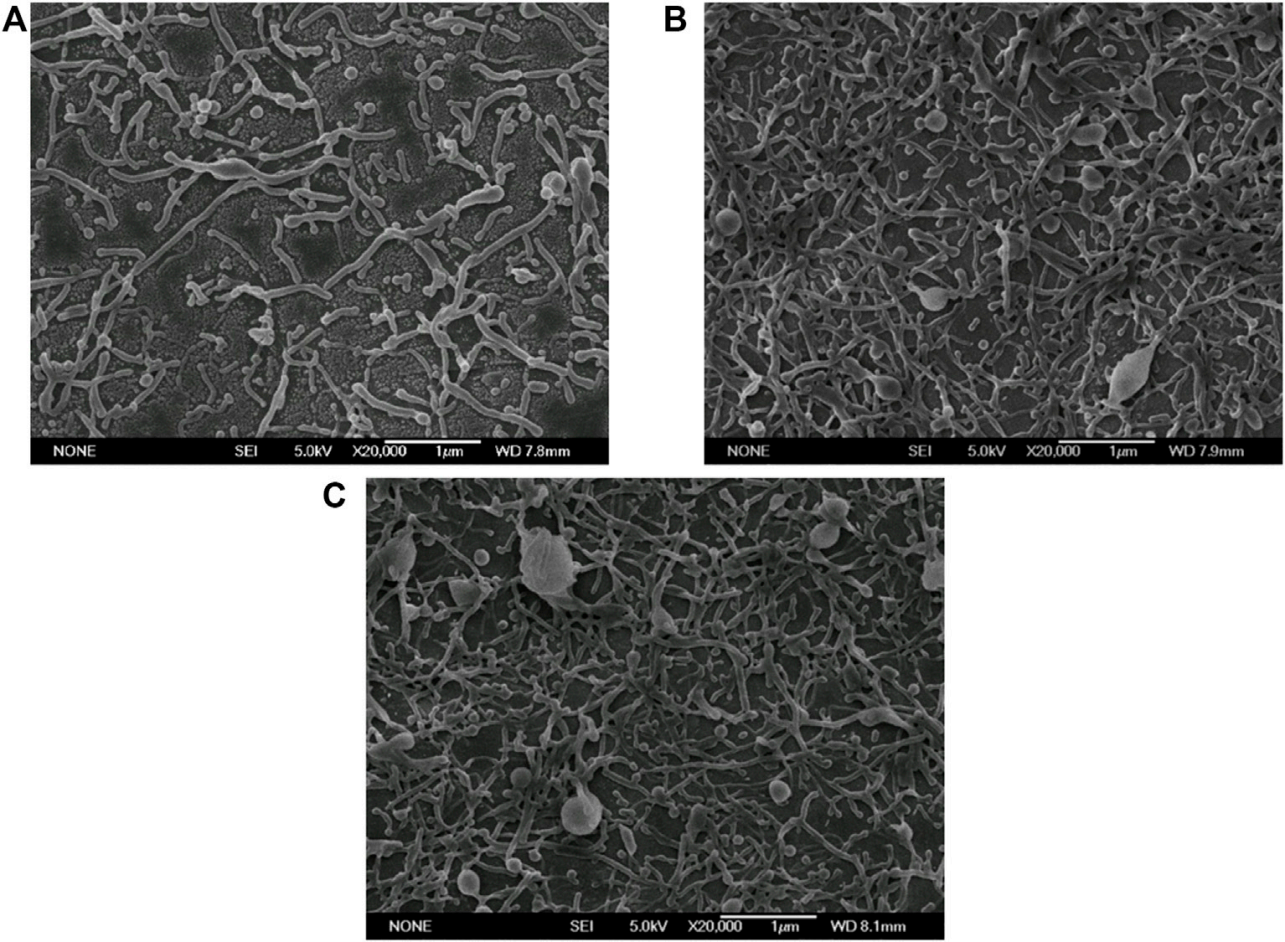
Electron micrographs of PLGA nanoparticles prepared at different oil phase volumes (Scale bar: 1 μm). **(A)** .5 ml; **(B)** 1.0 ml; **(C)** 1.5 ml.

The experiment investigated different oil phase volumes at the PLGA concentration of 20 mg/ml, which were .5, 1.0, and 1.5 ml, respectively. As shown in Figure 5, it seemed that the oil volume had no obvious effect on the particle size and deformation. Only a few near spherical particles occurred when the higher oil volume (1.0 and 1.5 ml) was used. The uniformity of initial droplet size was a key factor to determine the uniformity of nanorods. When the emulsification conditions (ultrasonic power) were the same, the initial droplets obtained with small volume of oil phase volume were relatively uniform. When the volume of the oil phase increased, the insufficient ultrasonic emulsification might lead to uneven initial droplets, and finally, the nanorods also deformed unevenly.

#### 3.2.6 Effect of PVA concentration

As mentioned above in the beginning of Section 3.2, the contradiction between initial droplet size and droplet deformation can be balanced by changing from one-step method to two-step method. Therefore, there was two PVA concentrations in the two-step ESE method, which was thePVA 1 concentration for particle formation and the PVA 2 concentrationfor deforming and stretching.

**FIGURE 6:**
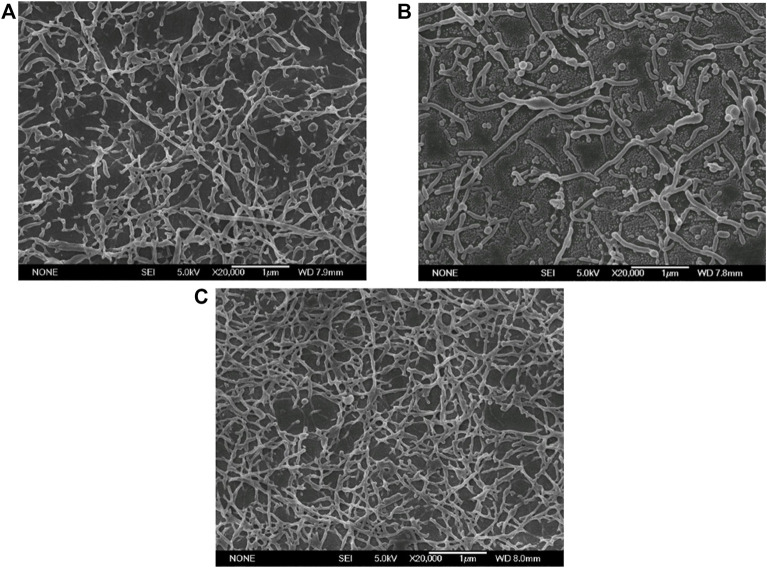
Electron micrographs of PLGA nanoparticles prepared with different PVA 1 concentrations (Scale bar: 1 μm).**(A)** .1 wt%; **(B)** .5 wt%; **(C)** 1.0 wt%.

First of all, The influence of three different levels (.1 wt%, .5 wt%, and 1.0 wt%) of the PVA 1concentration was explored on the deformation and yields of nanoparticles. As shown in Figure 6, there was no significant difference in the morphology among the nanoparticles fabricated by different concentration of the PVA 1. While, nanorods with the lowest PVA 1 concentration showed a little bit thinner minor axis than other groups. But many ultrasmall nanospheres could be found in this group. This phenomenon was probably due to the unstable droplets formed at this very low concentration of PVA 1 concentration. Some droplets could be broken up again during the emulsification and solidification. In consideration of the stability problem, the intermediate concentration of .5 wt% was used as the PVA 1 concentration in subsequent experiment.

**FIGURE 7:**
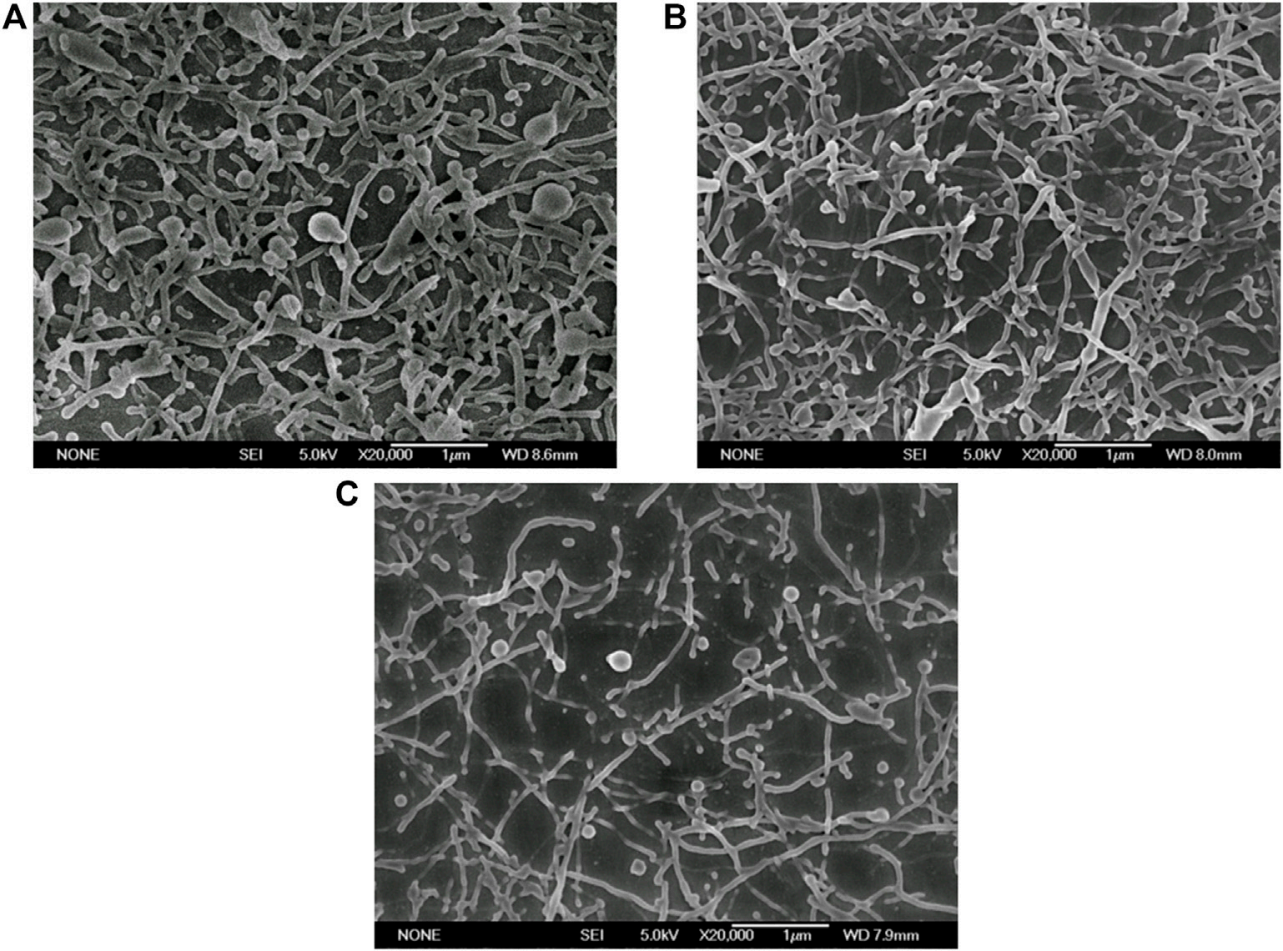
Electron micrographs of PLGA nanoparticles prepared with different high PVA concentrations (Scale bar: 1 μm) **(A)** 1.0 wt%; **(B)** 2.0 wt%; **(C)** 3.0 wt%.

Furthermore, the three different PVA 2 concentrations (1.0 wt%, 2.0 wt%, and 3.0 wt%) in the second step were investigated. As shown in Figure 7, nanoparticles prepared by different concentrations of PVA 2 showed little difference in the deformation ability. However, with the increase of the concentration of PVA, the size of minor axis was decreased, which could be attributed to the high Ca and low M values at high concentration of PVA 2 in the second step.

Overall, through the two-step ESE method and combined with Na_2_HPO_4_ as the deformation inducer and sonication as the emulsification technique, rod-shaped nanoparticles with the size in the nano range could be obtained. By using the single parameter optimization method, both the aspect ratio and the rod fabrication yield were increased. However, there were still some problems: 1) At the end of single factor optimization, there were still a few nanospheres fabricated with nanorods (Figure 7C), which could be due to that the single factor optimization could miss some important parameter and level combinations; 2) The significance of the studied parameters on the nanorods cannot be obtained through this single factor method. We cannot tell which parameter had the most powerful effect on the nanodroplets deformation; 3) It could be difficult to find the difference in size and shape between different levels through visual observation of the SEM images, thereby calling for the quantitative description. In order to solve these problem, the orthogonal experiment design combined with the quantitative measurement of the size and yield was adopted in following section to optimize the formulation of nanorods.

### 3.3 Optimization and preparation of PLGA nanorods by orthogonal test

**FIGURE 8:**
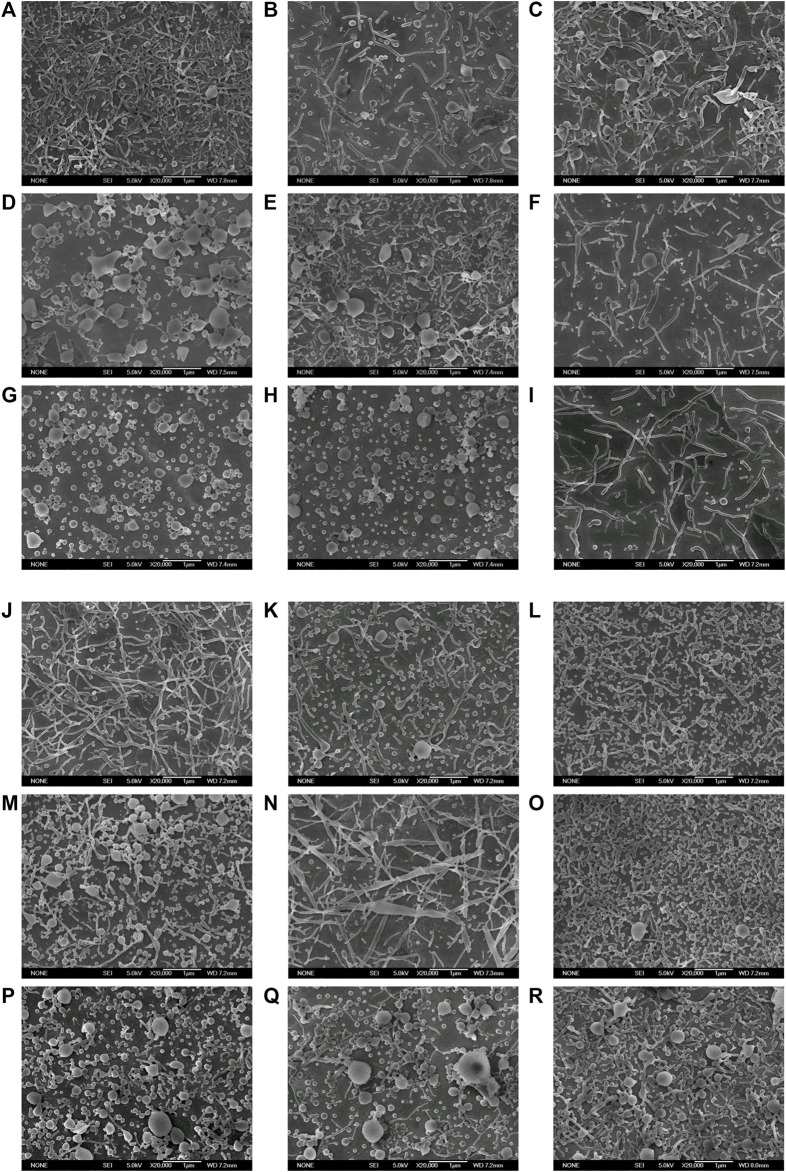
Electron micrographs of PLGA nanoparticles prepared with 18 orthogonally arranged tests for the six-factor three-level case (Scale bar: 1 μm). (A–R) represented prescriptions 1–18 in Supplementary Table S2 respectively.

Figure 8 displays the electron micrographs of PLGA nanoparticles prepared with 18 orthogonally arranged tests for the six-factor three-level case and Supplementary Tables S3, S4 demonstrate the important particle characteristics obtained from each run including the aspect ratio and the rod fabrication yield. Nanoparticles prepared at 18 different combinations of factors/levels demonstrated the aspect ratio between 1.14 ± .09 and 6.01 ± 2.29 (Supplementary Table S3) and the rod fabrication yield between 0% and 100% (Supplementary Table S4). And nanoparticle preparations resulted in a range of length between 67.8 ± 36.2 nm and 810.38 ± 321.88 nm (Supplementary Table S5), and width between 20.38 ± 9.64 nm and 286.50 ± 95.87 nm (Supplementary Table S6). The outcome of statistical analysis performed on each characteristic individually was also provided below.

The main effect plots for the aspect ratio versus different factor/level in Supplementary Table S3 shows that how aspect ratio was affected by factors at different levels. The difference between the maximum and minimum aspect ratio (*R* value) was highest in case of PVA 2 concentration (2.25), followed by PVA 1 concentration (1.66), Na_2_HPO_4_ concentration (1.50), PLGA concentration (1.27), oil volume (.19) and ultrasonic power (.06), which meant the PVA 2 concentration had the largest effect on aspect ratio, followed by PVA 1 concentration, Na_2_HPO_4_ concentration, PLGA concentration, oil volume and ultrasonic power.

**FIGURE 9:**
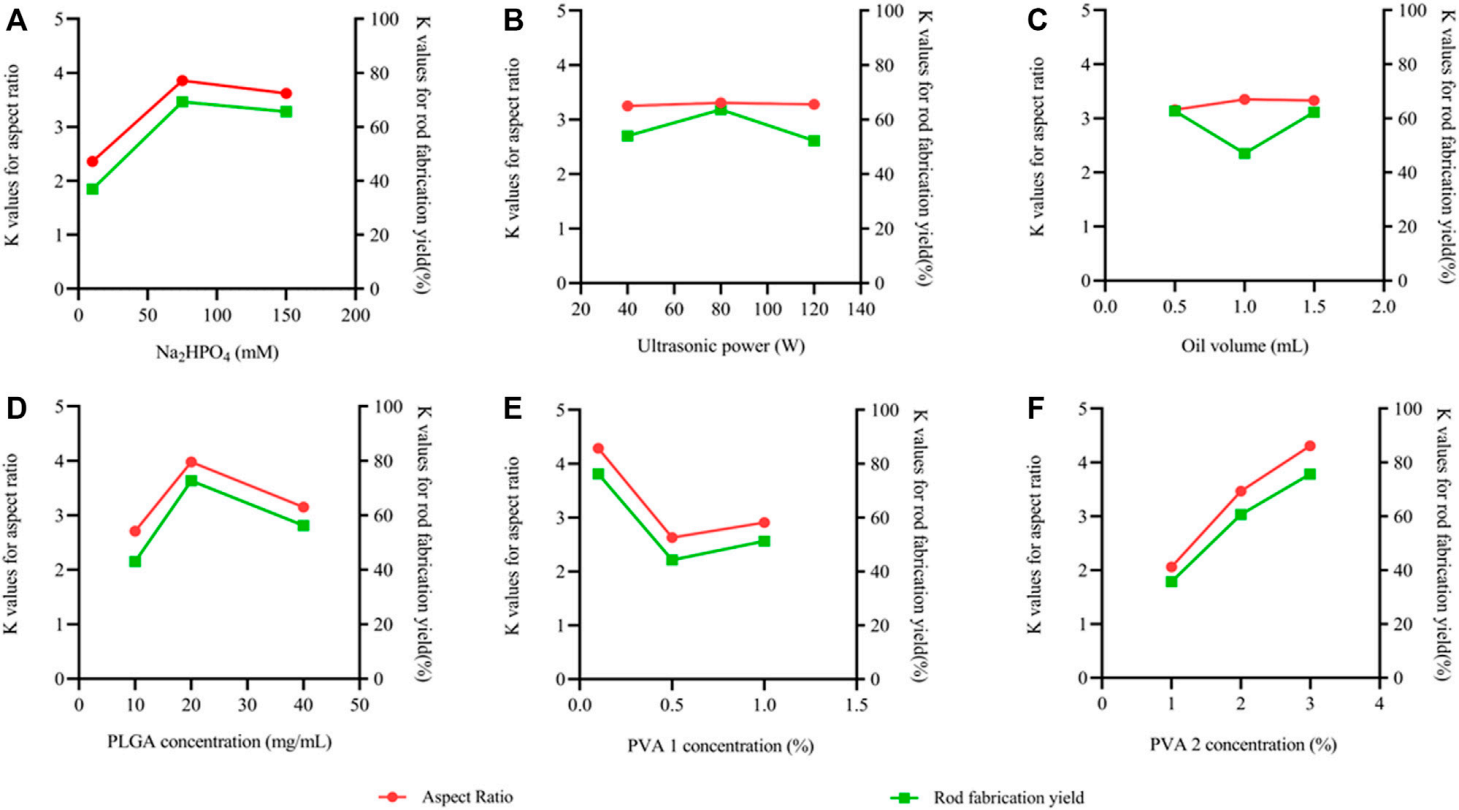
Main effect plots for the effects of the 6 parameters on the mean aspect ratio and the mean rod fabrication yield for the L18 orthogonally arranged design. The left vertical axis shows the mean aspect ratio, the right vertical axis shows the mean rod fabrication yield and the horizontal axis shows three levels (low, medium and high) of the parameters. **(A)** Na_2_HPO_4_ concentration, **(B)** Ultrasonic power, **(C)** Oil volume, **(D)** PLGA concentration, **(E)** PVA 1 concentration, **(F)** PVA 2 concentration.

Supplementary Table S4 demonstrates how the rod fabrication yield is changed with various factors/levels. PVA 2 concentration had the highest *R* value (40.00%) followed by Na_2_HPO_4_ concentration (32.33%), PVA 1 concentration (32.00%), PLGA concentration (29.67%), oil volume (15.67%), and ultrasonic power (11.33%). Accordingly, The three most important parameters affecting the rod fabrication yield were PVA 2 concentration, Na_2_HPO_4_ concentration and PVA 1 concentration, followed by oil volume, PLGA concentration and ultrasonic power. To gain insight into each factor’s effect and thereby to propose an optimal parameter combination, more detailed analysis had been carried out in the following sections. Figure 9 demonstrates the main effect plot for the aspect ratio and the rod fabrication yield versus factor/levels.

#### 3.3.1 Na_2_HPO_4_ concentration

As shown in Table 1, three levels of Na_2_HPO_4_ concentrations of 10, 75, and 150 mM were employed in the study. Figure 9A displays the K values of the aspect ratio and the rod fabrication yield on the left and right sidebar, respectively, corresponding to the three different levels of Na_2_HPO_4_ concentration. As can be clearly seen from the Figure 9A, with the increase of Na_2_HPO_4_ concentration, the aspect ratio and the rod fabrication yield increased; When the concentration exceeded 75 mM, they shown a slight downward trend. Thus, the Na_2_HPO_4_ concentration with 75 mM produced the best aspect ratio results, showing the best rod fabrication yield.

It was speculated that the Na_2_HPO_4_ molecule was in favor of forming hydrogen bonds with the carboxyl groups of PLGA, which induced the deformation of emulsion and resulted in the fabrication of non-spherical polymeric nano/micro-architectures. Similar results could be found in Ruifeng Li’s research team, who considered that the special chemical structure of STP might be in favor of inducing the formation of non-spherical polymeric micro-architectures and indicated that the hydrogen bonds formed between hydroxyl groups in PLGA and oxygen atoms in STP (Li et al., 2010). With the increase of Na_2_HPO_4_, the hydrogen bond interaction became strong so that the deformation was severe. Nevertheless, when the Na_2_HPO_4_ concentration exceeded 75 mM, the hydrogen bond interaction became saturation and then the competitive inhibition between molecules led to the decrease in the aspect ratio and nanorod fabrication yield.

#### 3.3.2 Ultrasonic power

The effects of the ultrasonic power on the aspect ratio and the rod fabrication yield are displayed in Figure 9B. The K value of the aspect ratio was between 3.25 and 3.31 in all of the studied ultrasonic power range, indicating that the ultrasonic power had little effect on the aspect ratio. Similarly, the K value of rod fabrication yield was between 52.33% and 63.67%, demonstrating that the ultrasonic power also had little effect on the deformability capacity of nanoparticles.

As is well-known, the ultrasonic power could affect the size of the initial emulsion droplets. In general, the initial droplet size decreased with increasing ultrasound power, which could lead to a low Ca and then hinder deformation. However, there was no obvious difference between the results of the aspect ratio and the rod fabrication yield on the three levels, which was probably due to that the ultrasonic power of the three levels had little effect on the initial size of the droplets.

#### 3.3.3 Oil voulme

Three levels of oil volume of .5, 1.0, and 1.5 ml had been employed in the tests, and their effects on the aspect ratio and rod fabrication yield were plotted in Figure 9C. It was observed that an oil volume of 1.0 ml produced the rod fabrication yield of 47.00%. The low and high oil volume produced the slightly higher yield of 62.67% and 62.33%, respectively. While, the aspect ratio was stable between 3.16 and 3.35 in the oil volume range of .5–1.5 ml.

This phenomenon could be explained by the balance between the interfacial tension and the initial droplet size. Due to the more hydrophobic nature of lactic acid (extra methyl side group) of PLGA, a higher interfacial tension often exist between the oil droplet and the continuous phase (Panyam et al., 2004). Therefore, when the concentration of PLGA in oil phase was constant, the amount of PLGA would increase with the volume of the oil. Thus, the interfacial tension between the oil droplet and the continuous phase also increased and the Ca value decreased, which could hinder the deformation and made the rod yield decreased. However, further increasing the oil volume to 1.5 ml could result in large initial droplets due to insufficient shearing, which could overcome the higher interfacial tension produced by PLGA organic phase and increase the Ca value. Thus, rod fabrication yield decreased first and then increased with the increase of the oil volume.

#### 3.3.4 PLGA concentration

Figure 9D displays obviously the effect of PLGA concentration on deformation. Increasing PLGA concentration corresponded to increase in the oil phase viscosity. The percentage of nanorods in the fabricated particles was 43%, 72.67%, and 56.33% for an oil phase with PLGA concentration of 10, 20, and 40 mg/ml, respectively. The corresponding average aspect ratios were 2.71, 3.98, and 3.15.

At a low PLGA concentration, droplets were easily broken up into small droplets, leading to a low *α* value and thereby decrease the Ca value to weaken deformation. So increasing the PLGA concentration could stabilize the droplets into a suitable size to increase Ca value and promote deformation. Nevertherless, further increasing the PLGA concentration to 40 mg/ml decreased the aspect ratio and rods yield, which could be explained by the increase in the viscosity ratio M to hinder the deformation.

#### 3.3.5 PVA concentration

The two-step method was set up with two different PVA concentrations of aqueous phase. Small-sized droplets are formed at PVA 1 concentration in the first step, followed by a second step of increasing the viscosity of continuous phase to enable the stretching of small droplets into rod particles.

As is shown in Figure 9E, the effect of PVA concentration on aspect ratio and yield was studied for PVA 1 concentration of .1 wt%, .5 wt%, and 1.0 wt% in the first step and PVA 2 concentration of 1.0 wt%, 2.0 wt%, and 3.0 wt% in the second step. In the results of the PVA 1 concentration (first step), the aspect ratio of nanoparticles had a tendency to decrease first and then increase when PVA concentration was increased from .1 wt% to 1.0 wt%, which were respecctively 2.06, 3.47, and 4.31. And the rod fabrication yield had same trend as the aspect ratio, which were respectively 76.33%, 44.33%, and 51.33%.

According to the above results, the PVA 1 concentration in the first step was more favorable for the deformation. This was because the PVA 1 concentration could help produce large droplets (high α value), which was beneficial to the high Ca and then the deformation. When the PVA concentration was from .5 wt% to 1.0 wt%, there was very slight increase in the aspect ratio and rods yield because the initial droplets size was reduced to its limit under sonication but the viscosity of the final continuous phase might increase modestly with the help of the increased PVA amount in the first step.

Furthermore, the more significant impact of PVA 2 concentration (second step) on the aspect ratio and the rod fabrication yieldcan be observed from Figure 9F, in which the K values of increased with the increase of PVA concentration. An increase in PVA concentration from 1.0 wt% to 3.0 wt% made the K values of the aspect ratio increase from 2.06 to 4.31, and the K values of the rod fabrication yield increased from 35.67% to 75.67%. It was not difficult to clarify the mechanism behind it. When the PVA 2 concentration in the second step was increased, the viscosity of aqueous phase increased, which produced a low M value and the particles were easily stretched into rod-shaped nanoparticles.

#### 3.3.6 Optimal parameters based on the results of orthogonally designed experiments

Taken collectively the results above, we could propose the optimal combination of parameters to preparate nanorods with high aspect ratio and rods fabrication yield. Overall, fabrication conditions for an optimized PLGA nanorods formulation were determined in our system as:

Na_2_HPO_4_ concentration: 75 mM (Level 2)
Ultrasonic Power: 80 W (Level 2)
Oli Volume: .5 ml (Level 1)
PLGA Concentration: 20 ml (Level 2)
PVA 1 Concentration in the first step: .1 wt% (Level 1)/1.0 wt% (level 3)
PVA 2 Concentration in the second step: 3.0 wt% (Level 3)

**FIGURE 10:**
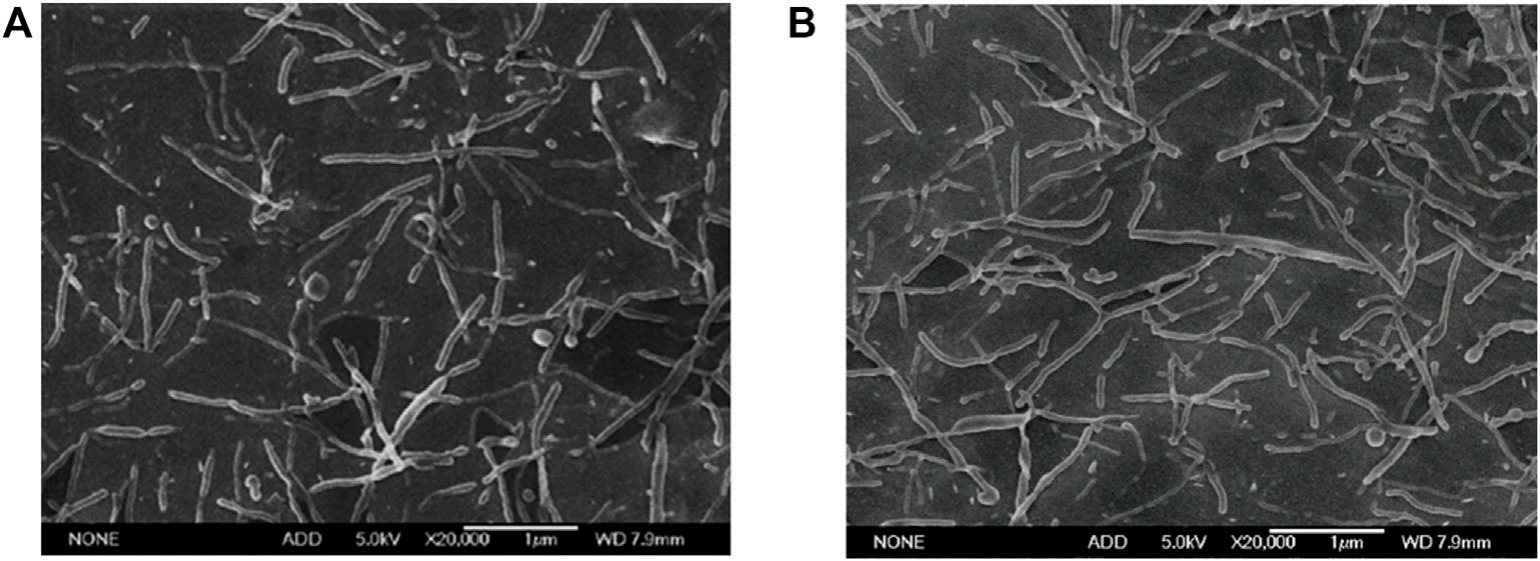
Electron micrographs of PLGA nanorods prepared with optimized formulation (Scale bar: 1 μm). **(A)** The PVA 1 concentration of .1 wt%; **(B)** the PVA 1 concentration of 1.0 wt%.

**FIGURE 11:**
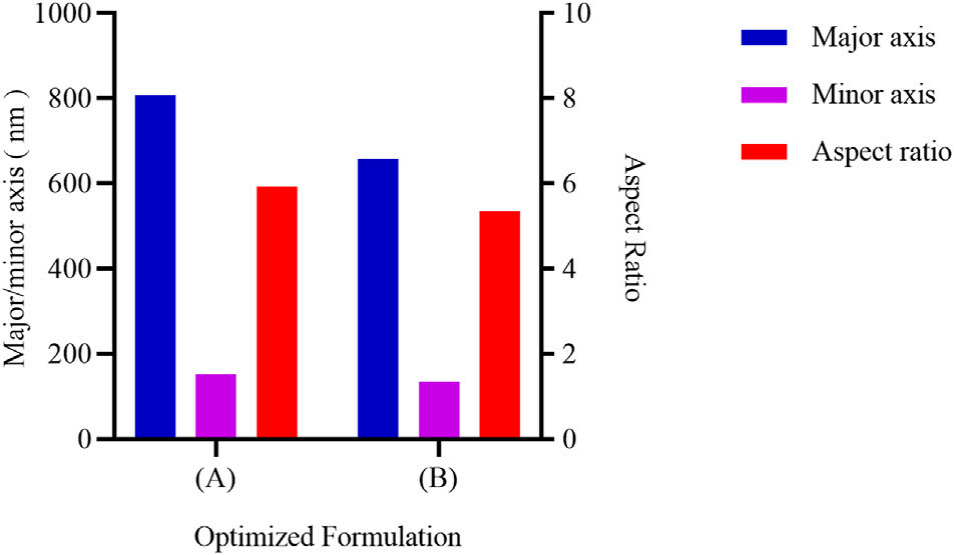
The size and aspect ratio of PLGA nanorods prepared with optimized formulation. The left vertical axis shows the mean major/minor axis, the right vertical axis shows the mean aspect ratio. **(A)** The PVA 1 concentration of .1 wt%; **(B)** The PVA 1 concentration of 1.0 wt%.

The blank PLGA nanorods based on the above prescriptions were prepared. Morphologies and characteristics of nanoparticles are shown in Figures 10, 11. The optimized fabrication method prepared PLGA nanorods with major axis of 808.01 ± 442.11/657.77 ± 307.63 nm, minor axis of 151.72 ± 88.44/135.49 ± 72.66 nm, and aspect ratio of 5.93 ± 2.39/5.35 ± 2.05. The minor axis was down to 150 nm, which was significantly lower than the research of Omolola Safari et al. (2018), who had prepared the PLGA nanorods with the minor axis down to 700 nm. To the best of our knowledge, this was the first time to successfully prepare the PLGA nanorods in nano size range by the ESE method. Compared with other method for constructing polymeric nanorods such as the film stretching technique (Kapate et al., 2021), our modified ESE method was simple, cheap, easy to scale up and did not need special instruments and harsh conditions. Moreover, the ESE method had been widely used to encapsulate hydrophilic and hydrophobic drugs. So, the nanorods prepared by the modified ESE method might also have great potential in application in drug delivery system.

### 3.4 Preparation and characterization of the paclitaxel loaded PLGA nanorods

**FIGURE 12:**
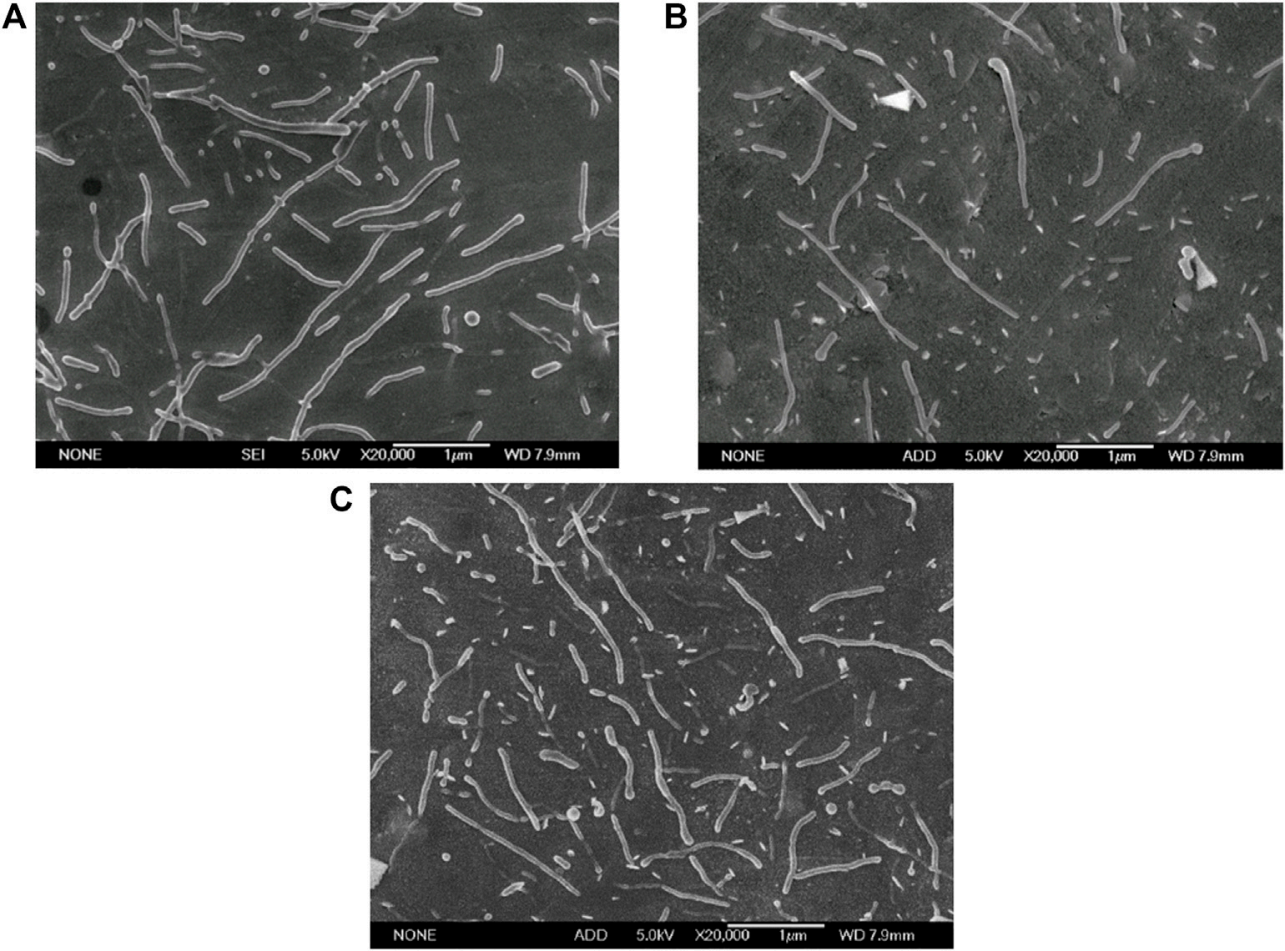
Electron micrographs of PTX loaded PLGA nanorods prepared with optimized formulation in different PTX concentrations (Scale bar: 1 μm). **(A)** 4% PTX; **(B)** 5% PTX; **(C)** 6% PTX.

The anti-cancer drug paclitaxel was added in the oil phase as the model drug to investigate the feasibility of our method for preparing drug loaded nanorods. Three paclitaxel concentrations of 4%, 5%, and 6% (percentage of the weight of paclitaxel in the PLGA particles) were used respectively and the results are represented in Figure 12 and Table 2.

**TABLE 2:** Characterization of the paclitaxel-loaded PLGA nanorods prepared with different PTX concentration.

PTX conc. (%)	Major axis		Minor axis		Aspect ratio		LE%	EE%
	Mean (nm)	SD	Mean (nm)	SD	Mean	SD		
4	809.74	325.02	178.62	56.57	4.75	1.90	1.78	44.43
5	993.48	434.48	182.11	65.94	5.60	1.84	2.55	51.04
6	913.70	279.66	204.26	63.73	4.69	1.39	4.26	71.03

^a^ Conc, concentration.

The results showed that the optimized fabrication method produced paclitaxel loaded PLGA nanorods with major axis of 809.74 ± 325.02, 993.48 ± 434.48, and 913.70 ± 279.66, with minor axis of 178.62 ± 56.57, 182.11 ± 65.94, and 204.26 ± 63.73 nm, and with aspect ratio of 4.75 ± 1.90, 5.60 ± 1.84, and 4.69 ± 1.39 at the PTX concentration of 4%, 5%, 6% (Table 2). The drug loading rate and encapsulation efficiency of paclitaxel loaded PLGA nanorods also showed an upward trend with the increase of the PTX concentration. The highest LE and EE investigated in the present study can achieve 4.26% and 71.03% (Table 2), demonstrating that our method could effectively encapsulte drugs without changing the size and shape of the nanorods. The minor axis of the paclitaxel loaded PLGA rods was down to 180 nm, which is smaller than 200 nm, indicating the potential application of nanorods for targeted cancer therapy. In our future research, the anti-cancer efficacy and the *in vivo* benefit of the prepared paclitaxel loaded nanorods will be thoroughly investigated.

## 4 Conclusion

Non-spherical geometries were increasingly considered as an important factor in the design of drug delivery carriers. Here, the rod-shaped PLGA particles in the nano size range were successfully prepared using the two-step emulsion‐solvent evaporation (ESE) method combined with the guest molecule Na_2_HPO_4_ and sonication technique. It was shown that the size, shape and aspect ratio of these non-spherical nanoparticles could be controlled by careful manipulation of key process parameters. The orthogonal experimental design could help investigate the influence of parameters on the aspect ratio and rod fabrication yield and find the optimal formulation. The data showed that surfactant PVA concentration in the second step was the most important influential factor and the high concentration of PVA 2 was beneficial to the deformation. Detailed mechanism under the deformation was also discussed thoroughly. Finally, paclitaxel was successfully encapsulated into the nanorods. Overall, the modified ESE method for fabricating nanorods had advantages of simplicity in setup, small size in nano range, high nanorod yield and adaptability to different biodegradable polymers and therapeutics. In summary, our results not only enrich the ESE technique for preparing rod-shaped PLGA particles in nano size range, but also envision the potential application of nanorods for targeted cancer therapy with the delivery of paclitaxel.

## Data Availability

The original contributions presented in the study are included in the article/Supplementary Material, further inquiries can be directed to the corresponding authors.
