# Supplementary material for: Preparation and optimization of poly (lactic-co-glycolic acid) rod-shaped particles in nano size range for paclitaxel delivery
Source: Front Bioeng Biotechnol. 2022 Dec 16;10:1103990. doi: 10.3389/fbioe.2022.1103990 (PMC9800425; doi:10.3389/fbioe.2022.1103990)
Supplement: Supplementary file 1 [file DataSheet1.docx]

Supplementary Material

**Preparation and optimization of** **poly (lactic-co-glycolic acid) rod-shaped particles in nano size range for paclitaxel delivery**

**Mengyao Xu ^1,2†^, Zuyue Liao**^1^**^,2†^, Yang, Liu**^1^**^,2†^, Shiwei Guo**^1^**^,2^, Haiyang Hu**^1^**^,2^ , Tao Chen**^1^**^,2^, Yuesong Wu**^1^**^,2^, Shengli Wan**^1^**^,2^, Meiling Zhou**^1^**^,2^, Muhe Lu**^2^**, Shiluo Jiluo**^2^**, Lan Yao**^1^**, Xiaofeng Pu**^1^**^,^ Shurong Wang**^1^**^,2^***, **Qingze Fan**^1^**^,2^***

^1^ Department of Pharmacy, the Affiliated Hospital of Southwest Medical University, Luzhou, Sichuan, China, 646099.

^2^ Department of Clinical Pharmacy, School of Pharmacy, Southwest Medical University, Luzhou, Sichuan, China, 646099.

†These authors contributed equally to this work.

*Corresponding author:

Qingze Fan, Email: [qingzefan017@swmu.edu.cn](mailto:qingzefan017@swmu.edu.cn); Shurong Wang, Email: wangshurong011@swmu.edu.cn

1. **Supplementary Figures**

**
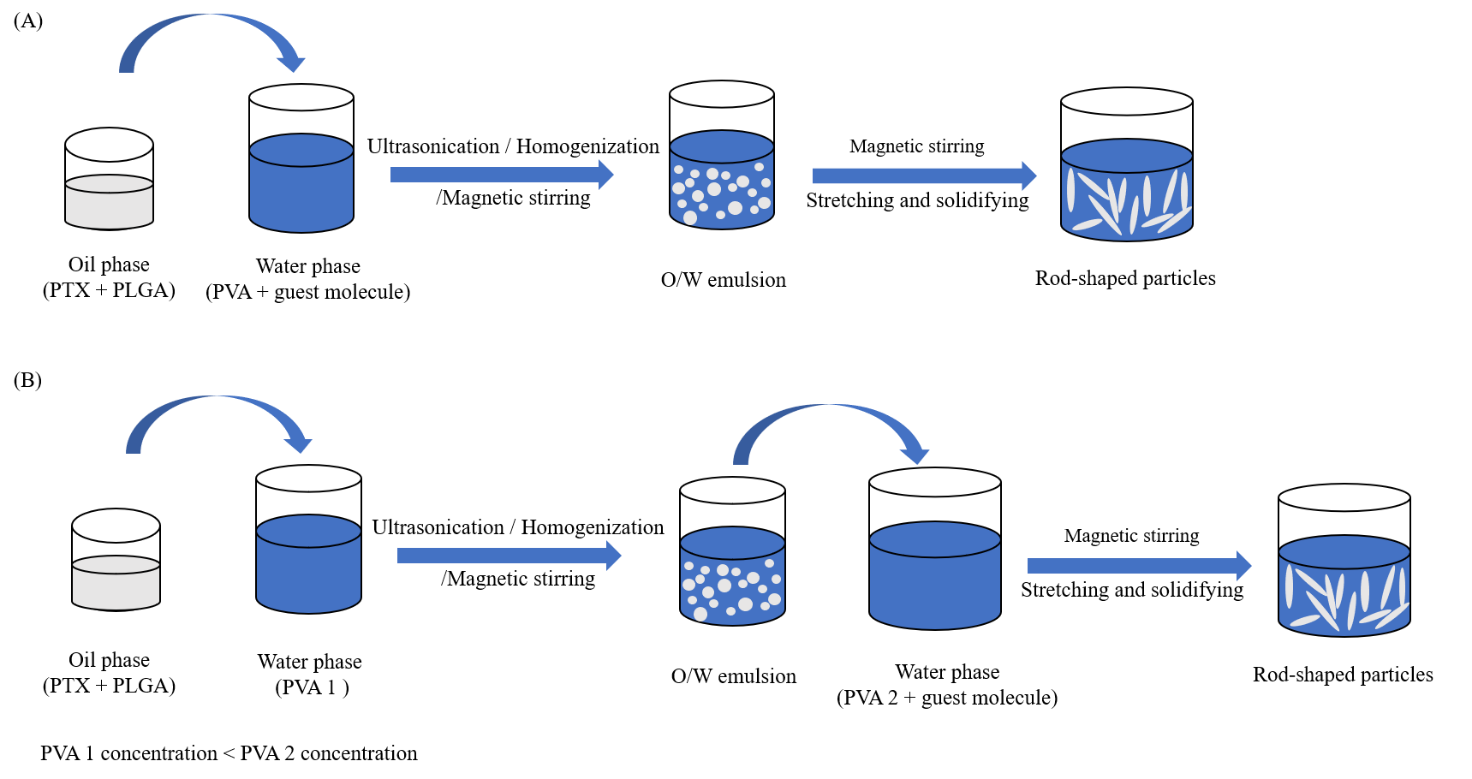
**

Figure S1. The flow chart for preparation of rod-shaped particles. (A) One-step method; (B) Two-step method.


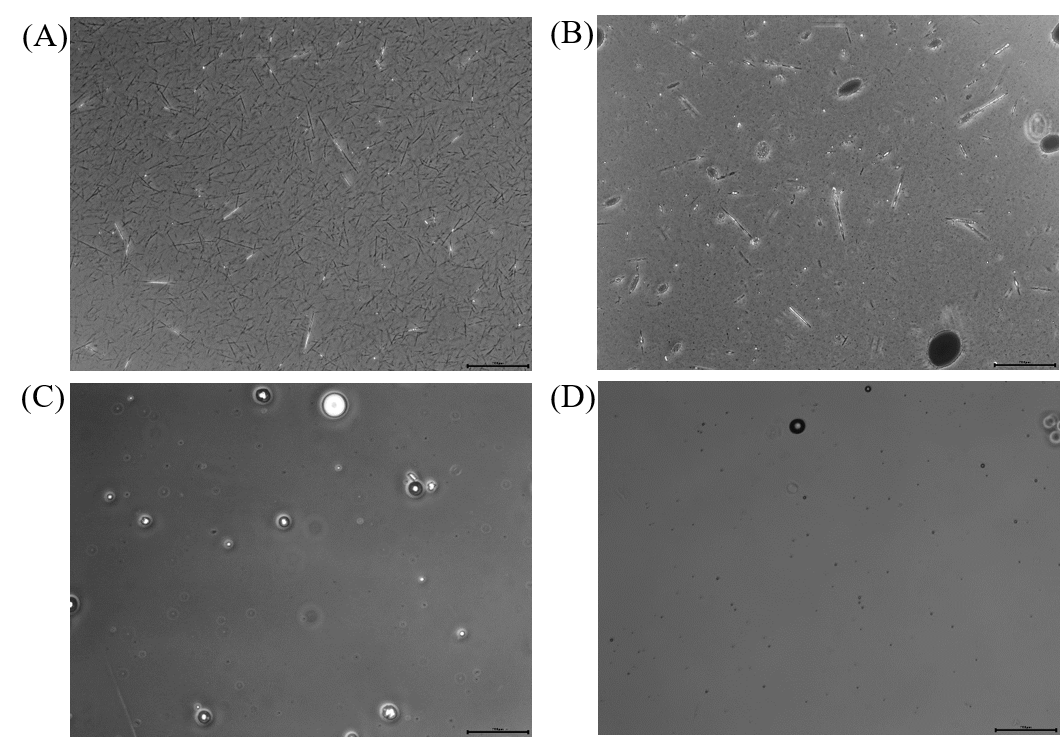


Figure S2. Effect of main components of PBS on deformation of PLGA microparticles under optical microscope (Scale bar: 200μm). (A) PBS; (B) Na_2_HPO_4_; (C) NaH_2_PO4; (D) NaCl. This experiment was performed by one-step ESE fabrication method


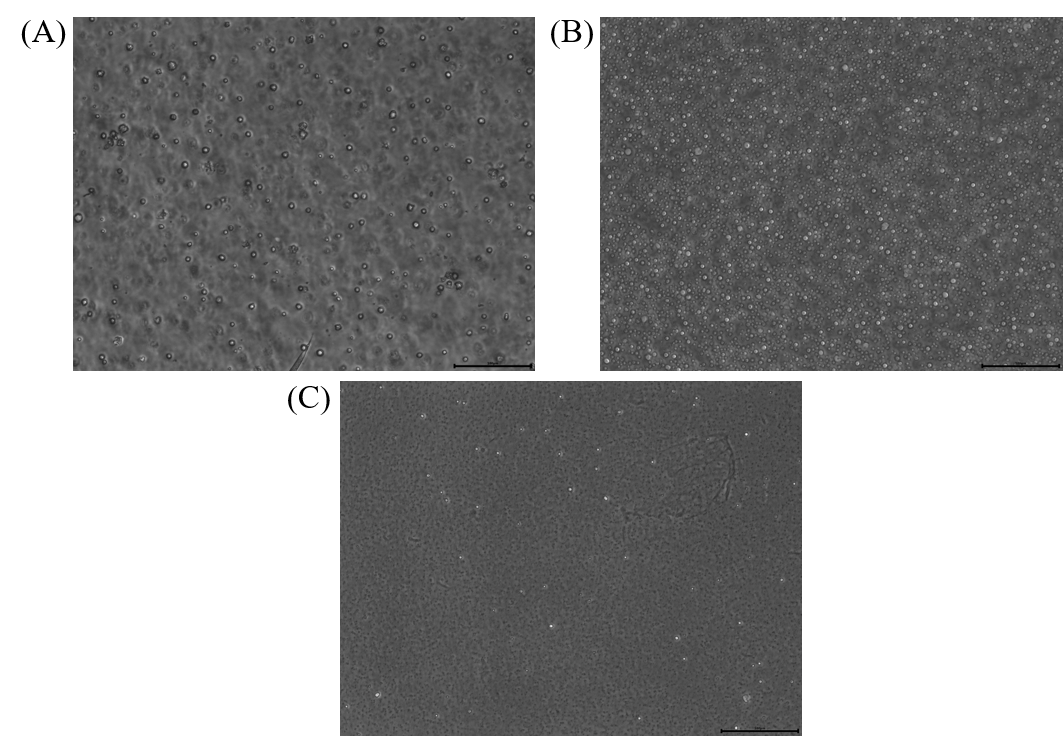


Figure S3. Effect of emulsification mode on the particle size of PLGA microparticles under light microscope (Scale bar: 100μm). (A) Magnetic stirring; (B) Homogenization; (C) Ultrasonication. This experiment was performed by one-step ESE fabrication method

1. **Supplementary Tables**

| Run# | Emulsification method | Ultrasonic power (W) | Na_2_HPO_4_ conc.(mM) | Oil volume (mL) | | PLGA conc.  (mg/mL) | | PVA 1 conc.  (wt%) | | PVA 2 conc.  (wt%) |
| --- | --- | --- | --- | --- | --- | --- | --- | --- | --- | --- |
| 1 | Ultrasonication | 120 | 150 | 0.5 | 20 | | 0.5 | | | 1.5 |
|  | Homogenization |  |  |  |  |  |  |  |  |  |
|  | Magnetic stirring |  |  |  |  |  |  |  |  |  |
| 2 | Ultrasonication | 40 | 150 | 0.5 | 20 | | 0.5 | | 1.5 | |
|  |  | 80 |  |  |  |  |  |  |  |  |
|  |  | 120 |  |  |  |  |  |  |  |  |
|  |  | 160 |  |  |  |  |  |  |  |  |
| 3 | Ultrasonication | 120 | 10 | 0.5 | 20 | | 0.5 | | 1.5 | |
|  |  |  | 50 |  |  |  |  |  |  |  |
|  |  |  | 100 |  |  |  |  |  |  |  |
|  |  |  | 150 |  |  |  |  |  |  |  |
| 4 | Ultrasonication | 120 | 150 | 0.5 | 20 | | 0.1 | | 1.5 | |
|  |  |  |  | 1.0 |  |  |  |  |  |  |
|  |  |  |  | 1.5 |  |  |  |  |  |  |
| 5 | Ultrasonication | 120 | 150 | 0.5 | 10 | | 0.5 | | 1.5 | |
|  |  |  |  |  | 20 | |  |  |  |  |
|  |  |  |  |  | 40 | |  |  |  |  |
| 6 | Ultrasonication | 120 | 150 | 0.5 | 20 | | 0.1 | | 1.5 | |
|  |  |  |  |  |  |  | 0.5 | |  |  |
|  |  |  |  |  |  |  | 1.0 | |  |  |
|  |  |  |  |  |  |  | 1.5 | |  |  |
| 7 | Ultrasonication | 120 | 150 | 0.5 | 20 | | 0.5 | | 1.0 | |
|  |  |  |  |  |  |  |  |  | 2.0 | |
|  |  |  |  |  |  |  |  |  | 3.0 | |

Table S1. the single-factor experiment design for two-step ESE method to explore the influence of multi-factors on the deformation

^1^Conc: concentration

Table S2. Combination of six factors three levels of orthogonal experiment design studied in preparation of PLGA nanorods L_18_ (3^6^) Array

| Run# | A^1^ | B^2^ | C^3^ | D^4^ | E^5^ | F^6^ |
| --- | --- | --- | --- | --- | --- | --- |
|  | mM | W | mL | mg/mL | wt% | wt% |
| 1 | 75 | 80 | 1.0 | 20 | 0.1 | 2.0 |
| 2 | 150 | 40 | 1.0 | 40 | 1.0 | 2.0 |
| 3 | 10 | 120 | 1.5 | 20 | 0.1 | 2.0 |
| 4 | 10 | 120 | 0.5 | 40 | 1.0 | 2.0 |
| 5 | 10 | 80 | 1.5 | 40 | 0.5 | 3.0 |
| 6 | 75 | 40 | 1.5 | 40 | 0.1 | 3.0 |
| 7 | 10 | 40 | 1.0 | 20 | 0.5 | 1.0 |
| 8 | 10 | 80 | 1.0 | 10 | 1.0 | 3.0 |
| 9 | 75 | 40 | 0.5 | 20 | 1.0 | 3.0 |
| 10 | 150 | 120 | 0.5 | 20 | 0.5 | 3.0 |
| 11 | 150 | 80 | 0.5 | 40 | 0.1 | 1.0 |
| 12 | 75 | 80 | 0.5 | 10 | 0.5 | 2.0 |
| 13 | 75 | 120 | 1.5 | 10 | 1.0 | 1.0 |
| 14 | 150 | 120 | 1.0 | 10 | 0.1 | 3.0 |
| 15 | 10 | 40 | 0.5 | 10 | 0.1 | 1.0 |
| 16 | 75 | 120 | 1.0 | 40 | 0.5 | 1.0 |
| 17 | 150 | 40 | 1.5 | 10 | 0.5 | 2.0 |
| 18 | 150 | 80 | 1.5 | 20 | 1.0 | 1.0 |

^1^A: Na_2_HPO_4_ concentration; ^2^B: ultrasonic power; ^3^C: oil volume; ^4^D: PLGA concentration; ^5^E: PVA 1 concentration in the first droplets formation step; ^6^F: PVA 2 concentration in the second deformation step.

Table S3. Range analysis for the effects of the 6 parameters on the aspect ratio for the L18 orthogonally arranged design.

| Run# | A^1^ | B^2^ | C^3^ | D^4^ | E^5^ | F^6^ | Aspect Ratio | | |
| --- | --- | --- | --- | --- | --- | --- | --- | --- | --- |
|  | mM | W | mL | mg/mL | wt% | wt% | mean | SD | |
| 1 | 75 | 80 | 1.0 | 20 | 0.1 | 2.0 | 6.01 | 2.29 | |
| 2 | 150 | 40 | 1.0 | 40 | 1.0 | 2.0 | 4.53 | 2.66 | |
| 3 | 10 | 120 | 1.5 | 20 | 0.1 | 2.0 | 4.07 | 1.84 | |
| 4 | 10 | 120 | 0.5 | 40 | 1.0 | 2.0 | 1.49 | 0.52 | |
| 5 | 10 | 80 | 1.5 | 40 | 0.5 | 3.0 | 3.94 | 2.01 | |
| 6 | 75 | 40 | 1.5 | 40 | 0.1 | 3.0 | 5.31 | 2.33 | |
| 7 | 10 | 40 | 1.0 | 20 | 0.5 | 1.0 | 1.14 | 0.09 | |
| 8 | 10 | 80 | 1.0 | 10 | 1.0 | 3.0 | 1.23 | 0.14 | |
| 9 | 75 | 40 | 0.5 | 20 | 1.0 | 3.0 | 4.88 | 1.89 | |
| 10 | 150 | 120 | 0.5 | 20 | 0.5 | 3.0 | 4.68 | 2.27 | |
| 11 | 150 | 80 | 0.5 | 40 | 0.1 | 1.0 | 2.24 | 1.18 | |
| 12 | 75 | 80 | 0.5 | 10 | 0.5 | 2.0 | 3.36 | 1.58 | |
| 13 | 75 | 120 | 1.5 | 10 | 1.0 | 1.0 | 2.25 | 1.34 | |
| 14 | 150 | 120 | 1.0 | 10 | 0.1 | 3.0 | 5.83 | 3.30 | |
| 15 | 10 | 40 | 0.5 | 10 | 0.1 | 1.0 | 2.30 | 1.19 | |
| 16 | 75 | 120 | 1.0 | 40 | 0.5 | 1.0 | 1.37 | 0.79 | |
| 17 | 150 | 40 | 1.5 | 10 | 0.5 | 2.0 | 1.32 | 0.77 | |
| 18 | 150 | 80 | 1.5 | 20 | 1.0 | 1.0 | 3.10 | 1.58 | |
| K_1_ | 2.36 | 3.25 | 3.16 | 2.71 | 4.29 | 2.06 |  |  |  |
| K_2_ | 3.86 | 3.31 | 3.35 | 3.98 | 2.63 | 3.47 |  |  |  |
| K_3_ | 3.62 | 3.28 | 3.33 | 3.15 | 2.91 | 4.31 |  |  |  |
| R | 1.50 | 0.06 | 0.19 | 1.27 | 1.66 | 2.25 |  |  |  |

^1^A: Na_2_HPO_4_ concentration; ^2^B: ultrasonic power; ^3^C: oil volume; ^4^D: PLGA concentration; ^5^E: PVA 1 concentration in the first droplets formation step; ^6^F: PVA 2 concentration in the second deformation step.

Table S4. Range analysis for the effects of the 6 parameters on the rod fabrication yield for the L18 orthogonally arranged design.

| Run# | A^1^ | B^2^ | C^3^ | D^4^ | E^5^ | F^6^ | Rod Yield |
| --- | --- | --- | --- | --- | --- | --- | --- |
|  | mM | W | mL | mg/mL | wt% | wt% |  |
| 1 | 75 | 80 | 1.0 | 20 | 0.1 | 2.0 | 96% |
| 2 | 150 | 40 | 1.0 | 40 | 1.0 | 2.0 | 90% |
| 3 | 10 | 120 | 1.5 | 20 | 0.1 | 2.0 | 82% |
| 4 | 10 | 120 | 0.5 | 40 | 1.0 | 2.0 | 10% |
| 5 | 10 | 80 | 1.5 | 40 | 0.5 | 3.0 | 84% |
| 6 | 75 | 40 | 1.5 | 40 | 0.1 | 3.0 | 94% |
| 7 | 10 | 40 | 1.0 | 20 | 0.5 | 1.0 | 0% |
| 8 | 10 | 80 | 1.0 | 10 | 1.0 | 3.0 | 0% |
| 9 | 75 | 40 | 0.5 | 20 | 1.0 | 3.0 | 100% |
| 10 | 150 | 120 | 0.5 | 20 | 0.5 | 3.0 | 88% |
| 11 | 150 | 80 | 0.5 | 40 | 0.1 | 1.0 | 52% |
| 12 | 75 | 80 | 0.5 | 10 | 0.5 | 2.0 | 80% |
| 13 | 75 | 120 | 1.5 | 10 | 1.0 | 1.0 | 38% |
| 14 | 150 | 120 | 1.0 | 10 | 0.1 | 3.0 | 88% |
| 15 | 10 | 40 | 0.5 | 10 | 0.1 | 1.0 | 46% |
| 16 | 75 | 120 | 1.0 | 40 | 0.5 | 1.0 | 8% |
| 17 | 150 | 40 | 1.5 | 10 | 0.5 | 2.0 | 6% |
| 18 | 150 | 80 | 1.5 | 20 | 1.0 | 1.0 | 70% |
| K_1_ | 37.00% | 54.00% | 62.67% | 43.00% | 76.33% | 35.67% |  |
| K_2_ | 69.33% | 63.67% | 47.00% | 72.67% | 44.33% | 60.67% |  |
| K_3_ | 65.67% | 52.33% | 62.33% | 56.33% | 51.33% | 75.67% |  |
| R | 32.33% | 11.33% | 15.67% | 29.67% | 32.00% | 40.00% |  |

^1^A: Na_2_HPO_4_ concentration; ^2^B: ultrasonic power; ^3^C: oil volume; ^4^D: PLGA concentration; ^5^E: PVA 1 concentration in the first droplets formation step; ^6^F: PVA 2 concentration in the second deformation step.

Table S5. Range analysis for the effects of the 6 parameters on the length for the L18 orthogonally arranged design.

| Run# | A^1^ | B^2^ | C^3^ | D^4^ | E^5^ | F^6^ | Length | |
| --- | --- | --- | --- | --- | --- | --- | --- | --- |
|  | mM | W | mL | mg/mL | % | % | mean | SD |
| 1 | 75 | 80 | 1.0 | 20 | 0.1 | 2.0 | 810.38 | 321.88 |
| 2 | 150 | 40 | 1.0 | 40 | 1.0 | 2.0 | 653.85 | 429.05 |
| 3 | 10 | 120 | 1.5 | 20 | 0.1 | 2.0 | 749.59 | 307.35 |
| 4 | 10 | 120 | 0.5 | 40 | 1.0 | 2.0 | 416.97 | 171.84 |
| 5 | 10 | 80 | 1.5 | 40 | 0.5 | 3.0 | 494.26 | 272.26 |
| 6 | 75 | 40 | 1.5 | 40 | 0.1 | 3.0 | 692.55 | 373.80 |
| 7 | 10 | 40 | 1.0 | 20 | 0.5 | 1.0 | 151.32 | 63.01 |
| 8 | 10 | 80 | 1.0 | 10 | 1.0 | 3.0 | 164.58 | 54.09 |
| 9 | 75 | 40 | 0.5 | 20 | 1.0 | 3.0 | 759.13 | 326.74 |
| 10 | 150 | 120 | 0.5 | 20 | 0.5 | 3.0 | 797.89 | 392.43 |
| 11 | 150 | 80 | 0.5 | 40 | 0.1 | 1.0 | 302.48 | 197.93 |
| 12 | 75 | 80 | 0.5 | 10 | 0.5 | 2.0 | 101.61 | 67.96 |
| 13 | 75 | 120 | 1.5 | 10 | 1.0 | 1.0 | 436.24 | 248.07 |
| 14 | 150 | 120 | 1.0 | 10 | 0.1 | 3.0 | 114.11 | 73.54 |
| 15 | 10 | 40 | 0.5 | 10 | 0.1 | 1.0 | 67.81 | 36.21 |
| 16 | 75 | 120 | 1.0 | 40 | 0.5 | 1.0 | 266.18 | 179.08 |
| 17 | 150 | 40 | 1.5 | 10 | 0.5 | 2.0 | 237.02 | 214.82 |
| 18 | 150 | 80 | 1.5 | 20 | 1.0 | 1.0 | 374.07 | 172.26 |
| K_1_ | 340.76 | 426.95 | 407.65 | 186.90 | 456.15 | 266.35 |  |  |
| K_2_ | 511.01 | 374.56 | 360.07 | 607.06 | 341.38 | 494.90 |  |  |
| K_3_ | 413.24 | 463.50 | 497.29 | 471.05 | 467.47 | 503.76 |  |  |
| R | 170.26 | 88.94 | 137.22 | 420.16 | 126.09 | 237.41 |  |  |

^1^A: Na_2_HPO_4_ concentration; ^2^B: ultrasonic power; ^3^C: oil volume; ^4^D: PLGA concentration; ^5^E: PVA 1 concentration in the first droplets formation step; ^6^F: PVA 2 concentration in the second deformation step.

Table S6. Range analysis for the effects of the 6 parameters on the width for the L18 orthogonally arranged design.

| Run# | A^1^ | B^2^ | C^3^ | D^4^ | E^5^ | F^6^ | Width | | |
| --- | --- | --- | --- | --- | --- | --- | --- | --- | --- |
|  | mM | W | mL | mg/mL | % | % | mean | SD | |
| 1 | 75 | 80 | 1.0 | 20 | 0.1 | 2.0 | 163.45 | 145.50 | |
| 2 | 150 | 40 | 1.0 | 40 | 1.0 | 2.0 | 150.83 | 79.10 | |
| 3 | 10 | 120 | 1.5 | 20 | 0.1 | 2.0 | 203.68 | 92.71 | |
| 4 | 10 | 120 | 0.5 | 40 | 1.0 | 2.0 | 286.50 | 95.87 | |
| 5 | 10 | 80 | 1.5 | 40 | 0.5 | 3.0 | 136.36 | 62.09 | |
| 6 | 75 | 40 | 1.5 | 40 | 0.1 | 3.0 | 135.17 | 61.18 | |
| 7 | 10 | 40 | 1.0 | 20 | 0.5 | 1.0 | 133.08 | 53.65 | |
| 8 | 10 | 80 | 1.0 | 10 | 1.0 | 3.0 | 134.73 | 44.07 | |
| 9 | 75 | 40 | 0.5 | 20 | 1.0 | 3.0 | 164.66 | 70.77 | |
| 10 | 150 | 120 | 0.5 | 20 | 0.5 | 3.0 | 190.51 | 91.44 | |
| 11 | 150 | 80 | 0.5 | 40 | 0.1 | 1.0 | 139.33 | 74.75 | |
| 12 | 75 | 80 | 0.5 | 10 | 0.5 | 2.0 | 30.43 | 11.16 | |
| 13 | 75 | 120 | 1.5 | 10 | 1.0 | 1.0 | 216.84 | 127.80 | |
| 14 | 150 | 120 | 1.0 | 10 | 0.1 | 3.0 | 20.38 | 9.64 | |
| 15 | 10 | 40 | 0.5 | 10 | 0.1 | 1.0 | 31.00 | 12.11 | |
| 16 | 75 | 120 | 1.0 | 40 | 0.5 | 1.0 | 196.30 | 89.47 | |
| 17 | 150 | 40 | 1.5 | 10 | 0.5 | 2.0 | 174.74 | 96.70 | |
| 18 | 150 | 80 | 1.5 | 20 | 1.0 | 1.0 | 135.98 | 57.43 | |
| K_1_ | 154.22 | 131.58 | 140.40 | 101.35 | 115.50 | 142.09 |  |  |  |
| K_2_ | 151.14 | 123.38 | 133.13 | 165.23 | 143.57 | 168.27 |  |  |  |
| K_3_ | 135.30 | 185.70 | 167.13 | 174.08 | 181.59 | 130.30 |  |  |  |
| R | 18.93 | 62.32 | 34.00 | 72.73 | 66.09 | 37.97 |  |  |  |

^1^A: Na_2_HPO_4_ concentration; ^2^B: ultrasonic power; ^3^C: oil volume; ^4^D: PLGA concentration; ^5^E: the concentration of the PVA 1in the first droplets formation step; ^6^F: the concentration of the PVA2 in the second deformation step.
